# A randomized, double-blinded, placebo-controlled clinical trial on Lactobacillus-containing cultured milk drink as adjuvant therapy for depression in irritable bowel syndrome

**DOI:** 10.1038/s41598-024-60029-2

**Published:** 2024-04-25

**Authors:** Marlynna Sarkawi, Raja Affendi Raja Ali, Norhazlina Abdul Wahab, Norshafila Diana Abdul Rathi, Norfilza Mohd Mokhtar

**Affiliations:** 1 Department of Physiology, Faculty of Medicine, Universiti Kebangsaan Malaysia, Jalan Yaacob Latif, Bandar Tun Razak, Cheras, 56000 Kuala Lumpur, Malaysia; 2 School of Medical and Life Sciences, Sunway University, Sunway City, 47500 Petaling Jaya, Malaysia; 3 GUT Research Group, Faculty of Medicine, Universiti Kebangsaan Malaysia, Kuala Lumpur, Malaysia; 4 Department of Basic Medical Sciences, Faculty of Medicine and Health Sciences, Universiti Malaysia Sarawak, 94300 Kota Samarahan, Malaysia

**Keywords:** Probiotics, Lactobacillus, Functional gastrointestinal diseases, Gut-brain axis, Irritable bowel syndrome, Microbiology, Gastroenterology, Health care

## Abstract

Irritable bowel syndrome (IBS) is frequently linked with coexisting mental illnesses. Our previous study discovered that 32.1% of IBS patients had subthreshold depression (SD), placing them at higher risk of developing major depression. Gut microbiota modulation through psychobiotics was found to influence depression via the gut-brain axis. However, the efficacy of lessening depression among IBS patients remains ambiguous. The study’s aim was to investigate the roles of cultured milk drinks containing 10^9^ cfu *Lactobacillus acidophilus* LA-5 and *Lactobacillus paracasei* L. CASEI-01 on depression and related variables among IBS participants with SD. A total of 110 IBS participants with normal mood (NM) and SD, were randomly assigned to one of four intervention groups: IBS-NM with placebo, IBS-NM with probiotic, IBS-SD with placebo, and IBS-SD with probiotic. Each participant was required to consume two bottles of cultured milk every day for a duration of 12 weeks. The following outcomes were assessed: depression risk, quality of life, the severity of IBS, and hormonal changes. The depression scores were significantly reduced in IBS-SD with probiotic and placebo from baseline (p < 0.001). Only IBS-SD with probiotic showed a significant rise in serotonin serum levels (p < 0.05). A significantly higher life quality measures were seen in IBS-SD with probiotic, IBS-SD with placebo, and IBS-NM with placebo (p < 0.05). All groups, both placebo and probiotic, reported significant improvement in IBS severity post-intervention with a higher prevalence of remission and mild IBS (p < 0.05). Dual strains *lactobacillus*-containing cultured milk drink via its regulation of relevant biomarkers, is a potential anti-depressive prophylactic agent for IBS patients at risk.

## Introduction

Presence of any form of psychological or psychiatric comorbidities among irritable bowel syndrome (IBS) patients are commonly reported across the globe^1,2^. Despite being classified as a functional gastrointestinal disorder (FGID) for many years, the emerging scientific findings supporting the bidirectional interaction of gut and brain has led to rebranding IBS as a disorder of gut-brain interaction (DGBI). The diagnosis of IBS relies on a symptoms-based criterion known as Rome IV, where individuals with IBS frequently present with recurrent abdominal pain accompanied by change in bowel habit or stool consistency appearing as either constipation or diarrhoea or both^3^. There are four subtypes of IBS that can affect an individual, including IBS-constipation (IBS-C), IBS-diarrhoea (IBS-D), IBS-mixed (IBS-M), and IBS-undetermined (IBS-U)^3^. IBS prevalence varies across regions with a global estimate ranging from 10 to 20%^4,5^. Prior research found that the prevalence of IBS was 10.9% and 14% respectively in the populations of the two states of Kelantan and Perak in Malaysia^5^.

Nowadays, IBS research on pathophysiology is focusing on the gut-brain crosstalk including immunological hyperactivation, visceral hypersensitivity, dysbiosis, disrupted intestinal permeability, and psychological causes^6,7^. The hypothesis of believing gut capability to regulate behaviour through brain functioning has been established in the early 20th Century^6^. Several studies have found the causal effect of psychological stress such as depression and anxiety, to triggering IBS-related gastrointestinal symptoms^3,8^. A higher prevalence of depression and anxiety was reported among IBS sufferers with approximately 30% have self-referred for medical consultations^2^. The hypothalamus–pituitary–adrenal (HPA) axis, as stress regulated pathway, is hyperactivated by psychological stress associated to academic examinations among university students, increasing their risk of developing IBS^9^.

According to a recent study, we identified that 32.1% of IBS patients had subthreshold depression, often referred to as subclinical or subsyndromal symptomatic depression^10^. Although there is no universally accepted definition for subthreshold depression, research clearly indicates that it is a risk factor for clinical depression, especially in younger populations^11^. In 2022, a subthreshold depression prevalence of 11.02% was reported, dominated by the population under the age of 18^12^. It was discovered that women are more prone to subthreshold depression than men. Without early intervention, subthreshold depression may escalate into major depressive disorder (MDD)^12^. They may be more likely to experience significant depression if they have any chronic illnesses, including IBS^13^.

Probiotics that were discovered by Elie Metchnikoff in the late nineteenth century have captured the interest of many researchers in recent years and are believed to modify the gut-brain axis in IBS patients with depression^14^. According to the International Scientific Association of Probiotics and Prebiotics (ISAPP), probiotics are defined as *“live microorganisms that, when administered in adequate amounts, confer a health benefit on the host”*^15^. People with MDD had low levels of *lactobacillus* and *bifidobacterium* strains, which was also observed in IBS subjects^16^. Moreover, the immunomodulatory effects of probiotic have been linked to anxiolytic and antidepressant outcomes in IBS patients and animal models^17,18^. An equilibrium between pro-inflammatory and anti-inflammatory cytokines is required to ameliorate IBS-like symptoms^19^. Our previous study also reported that 30-day cultured milk drink supplementation reduced intestinal transit time in IBS-C patients^20^. Probiotic supplements were just as effective as Duloxetine therapy at reducing the severity of depression symptoms in MDD patients^21^. However, probiotics were found to be more effective when used alongside with antidepressants such as SSRIs rather than as a sole therapy^22^. Additionally, a 16-week multi-strain probiotic therapy was found to be sufficient to improve patients' perspectives on their IBS-related quality of life (QOL) even after a 4-week wash-out period^23^. In our previous study, probiotics were found to have protective effects against chronic stress and depression by significantly lowering cortisol levels and activating the tryptophan hydroxylase enzyme to promote serotonin synthesis^24,25^. Therefore, the objective of this study was to evaluate the impact of probiotic-containing cultured milk drinks on gut-brain axis and to determine their effectiveness in reducing depression symptoms in IBS patients who are at risk particularly those with subthreshold depression.

## Methods

### Study design

**Figure 1:**
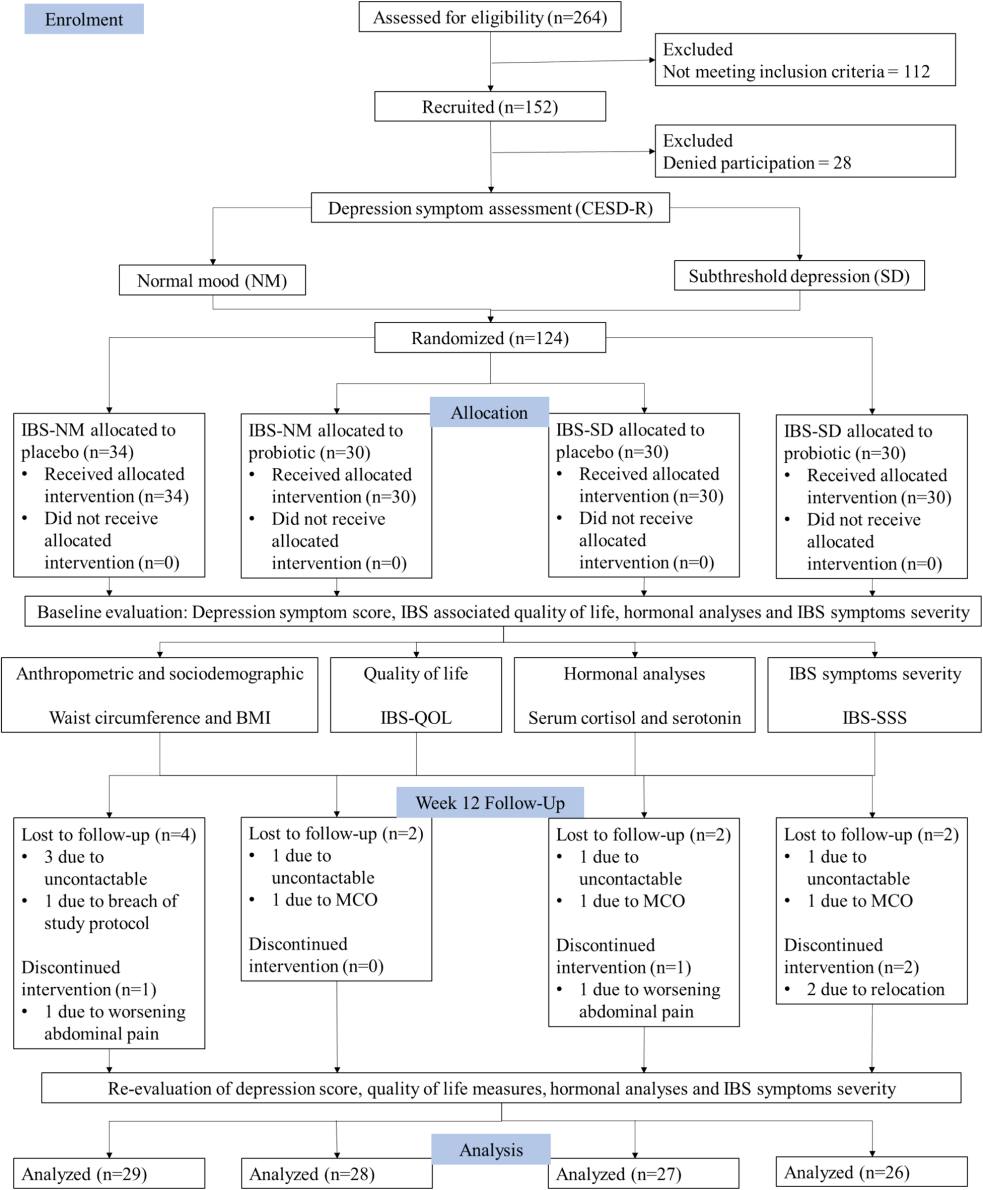
CONSORT diagram of the randomized control trial. *CESD-R* center for epidemiologic studies depression revised, *MCO* movement control order.

The disposition of participants is depicted in Fig. 1. This was a 12-week randomized, double-blinded, placebo-controlled, parallel clinical trial with IBS participants at the Universiti Kebangsaan Malaysia Medical Centre (UKMMC) from 22/11/2019 until 17/08/2022. The study protocol was approved by the institutional research ethics committee (Reference number UKM PPI.800-1/1/5/JEP-2019-312, initial approval 01/08/2019) and retrospectively registered under the International (US) Clinical Trial Registry (NCT05266443; First posted date on 04/03/2022). Informed consent was obtained from all participants prior to study enrolment and was performed in accordance with the Declaration of Helsinki. The participants were classified into two categories based on their depression symptoms score using the “*Center for Epidemiologic Studies Depression Revised*” (CESD-R) questionnaire: normal mood (NM) group (score < 16) and subthreshold depression (SD) group (score ≥ 16). Then, participants were randomly allocated to receive either a placebo or a probiotic-containing cultured milk drink, forming four intervention groups: IBS-NM placebo, IBS-NM probiotic, IBS-SD placebo, and IBS-SD probiotic. The outcomes were assessed at baseline and post-intervention.

### Sample size rationale

Sample size of 104 was required, calculated based on a prior study^26^ with a power of study of 0.8 and alpha error of 0.05. The main outcome of depression scores was chosen from the study, with mean difference of 11.1 and pooled standard deviation of 5.73 among patients receiving probiotic treatment^26^. Based on the sample size calculation, our study required 20 participants per group. The sample size acquired was increased by an additional 30% to account for dropouts based on previous study^27^ and the effect of the Covid-19 pandemic during the early recruitment phase. Therefore, a total participants of 124 were recruited to reduce the attrition bias resulted from Covid-19 related situations.

### Sample population

**Figure 2:**
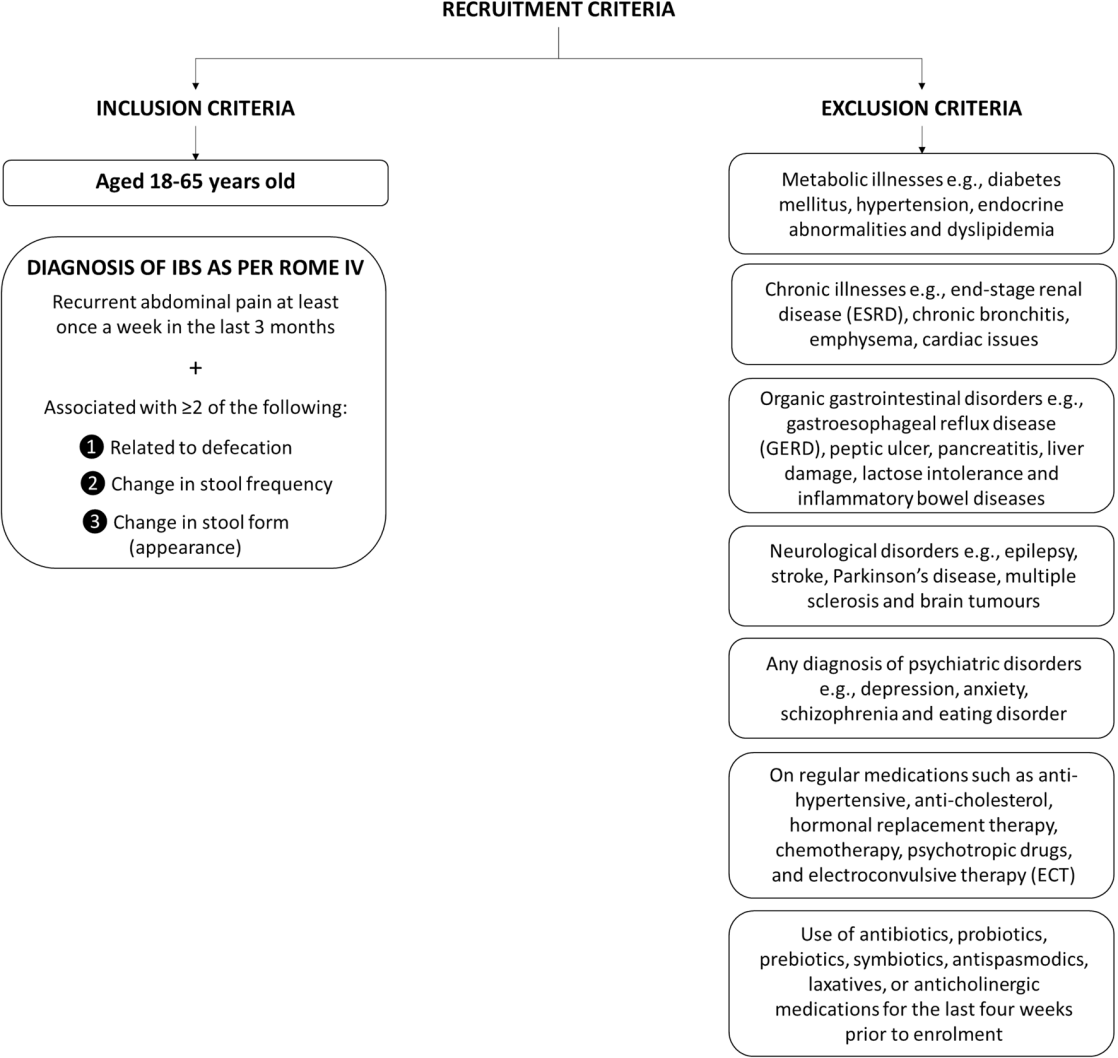
Inclusion and exclusion criteria for the study.

IBS participants, aged between 18 and 65, who met the Rome IV criteria^3^ were enrolled in the study (Fig. 2). Participants, including volunteers from the local community were evaluated for eligibility at the Gastroenterology clinic of UKMMC. Participants who did not fit the inclusion and exclusion criteria were eliminated from the study.

### Randomization and intervention

Participants in each group were randomly assigned to either a placebo or a probiotic-containing cultured milk drink using a simple randomization (1:1 ratio) for 12 weeks. The intervention products were packed in 125 ml plastic bottles with identical physical appearance. Each bottle consists of fructose, milk solids (non-fat), polydextrose, grape juice, glucose, stabilizer, and acid preservative as food conditioners, permitted flavoring and coloring. Meanwhile, the probiotic drink contains 1 × 10^9^ cfu of dual strain *Lactobacillus acidophilus*, LA-5 and *Lactobacillus paracasei*, L. PARACASEI-01. Each 125 ml of cultured milk drink has the following nutritional breakdown: 59 kcal of energy, 1.0 g of protein, 2.0 g of fibres, 12.6 g of carbohydrates, 9.6 g of total sugar (consisting of 7.4 g of fructose and 2.3 g of lactose). Participants were instructed to drink two bottles of cultured milk drinks daily. Both researchers and participants were blinded to the intervention and only disclosed upon trial completion.

### Baseline data

Data, such as height, weight, waist circumference and body mass index (BMI), was collected during the enrolment phase.

### Primary outcomes

The primary endpoint of this study was the change in depression symptoms score following the cultured milk drink intervention, assessed with *“Center for Epidemiologic Studies Depression Revised”* (CESD-R) questionnaire^28^. CESD-R is a validated self-reported questionnaire assessing individual’s depression symptoms through 20 items based on the American Psychiatric Association Diagnostic and Statistical Manual, version 5 (DSM-5)^29^. The total score of CESD-R is calculated based on the summation of the 20 items responses. The total score of CESD-R ranges between 0 and 60 points. Participants were divided into two groups: normal mood (CESD-R < 16) and subthreshold depression (CESD-R ≥ 16). Participants experiencing anhedonia or dysphoria for almost daily for two weeks with at least two to four symptoms of major depression outlined by DSM-5 were excluded from the study. Changes in depression symptom categories, such as subthreshold depression or normal mood, were also evaluated. Participants with serious depression symptoms were referred for further assessment with their consent.

### Secondary outcomes

Secondary outcomes include changes in the IBS-related QOL, gastrointestinal symptoms and hormonal analysis of cortisol and serotonin, with cultured milk drink intervention. Evaluation of the QOL perception and gastrointestinal symptoms severity utilises the *“Irritable Bowel Syndrome–Quality of Life”* (IBS–QOL) and *“Irritable Bowel Syndrome Severity Scoring System”* (IBS-SSS) questionnaires, respectively^30,31^. The total IBS-SSS scores was also used to determine the changes in the severity categories: remission (˂ 75), mild (75–174), moderate (175–299) and severe symptoms (≥ 300)^31^.

For hormonal analysis, the non-fasting serum samples were analyzed with competitive enzyme-linked immunosorbent assay (ELISA) (Elabscience) in accordance with standard calculation provided in the kit. Ten millilitres of blood were collected from each participant between 8 and 9 am, both before and after the intervention. In brief, an ELISA plate was loaded with standards and serum samples. The plate was incubated at 37 °C for 90 min. After incubation, 100μL Biotin-detection antibody working solution was added, and the plate was further incubated for 60 min in an incubator (dark environment, 37 °C). Plate was then undergoing three washings, and 100 μL HRP (horseradish peroxidase) conjugate working solution was added into each well. After 30 min of incubation at 37 °C, the plate was washed five more times using wash buffer and 90 μL of TMB (tetramethylbenzidine) substrate was added. The plate was incubated for 15–30 min for the formation of color. Stop solution was added accordance with to the kit instructions prior to reading. The standard curve was generated using cloud software (Myassay.com) that supports four-parametric logistic curve calculation. The data from ELISA was an average of duplicate readings. The biomarker level was calculated using the standards that came with the relevant ELISA kit.

### Compliance, adherence, and adverse effects (AE)

To ensure compliance and protocol adherence, strategies such as weekly reminder, scheduled delivery of interventional products and returning empty bottles upon delivery of new stock were implemented. Feedback on adverse events were gathered throughout the clinical trial. Affected participants were given appropriate techniques to lessen the negative effects before leaving the research. Participants were allowed to withdraw from the study voluntarily, or participants who experienced extreme abdominal pain, disruptive change in bowel motion, new diagnosis of gastroenterology, metabolic, or psychiatric illnesses, pregnancy, inability to comply with study protocol such as work relocation and consumption of prohibited products within the 12-week period were withdrawn from the study at any stage of the trial.

### Statistical analysis

The end-point data were analyzed using IBS SPSS Statistics, 28th version. The data distribution was assessed using the Kolmogorov–Smirnov normality test, skewness, and Kurtosis values. The normally distributed end-point data were expressed as mean and standard deviation, meanwhile the non-normally distributed data were expressed as median and interquartile range (IQR). Other categorical data were expressed using descriptive statistics such as frequency (n) and percentage (%). An independent t-test and Mann–Whitney U test was performed when comparing parameters between the NM and SD groups depending on the data distribution. One-way analysis of variance (ANOVA) or Kruskal–Wallis analysis was performed to compare the parameters between all groups at baseline according to the data distribution. The pre- and post-intervention data were compared with paired t-test for normally distributed data and Wilcoxon Signed-Rank test for the non-normally distributed data. The changes between baseline and end of trial were considered statistically significant with p < 0.05. Comparison of parameters at pre- and post-intervention between all groups was analysed using the General Linear Model (GLM) ANOVA for normally distributed data or Kruskal–Wallis for non-parametric analysis. Analysis of covariate (ANCOVA) was performed to compare the findings with baseline covariate adjustment. The effect size of intervention was determined from Cohen values (d coefficient) for normally distributed data.

### Ethical declarations

The study protocol was approved by the institutional research ethics committee of Universiti Kebangsaan Malaysia (UKM PPI.800-1/1/5/JEP-2019-312).

### Informed consent

Informed consent was obtained from all participants prior to study enrolment. All participants consented for the data to be published.

## Results

### Recruitment and follow-up

A total of 264 participants were evaluated for eligibility to participate in the study (Fig. 1). However, only 124 IBS participants who fulfilled the Rome IV criteria were enrolled and divided into normal mood and subthreshold depression groups. A total of 110 participants completed the 12-week trial. 14 participants were considered as dropouts due to non-compliance with the study protocol (n = 1), being uncontactable (n = 6), and unable to attend last visit (n = 3) due to Movement Control Order (MCO) enforced by the Malaysian government during early phase of Covid-19 outbreak. Another four participants were discontinued from the trial due to personal reasons (n = 2) and severe abdominal pain (n = 2).

### Baseline characteristics

The sociodemographic and anthropometric characteristics of enrolled participants are summarized in Table 1. At baseline, the CESD-R and the overall IBS-SSS scores, with two domains (bowel habit satisfaction and life interferences) were significantly higher in the IBS-SD group than the IBS-NM group (Supplementary Table 1S). The IBS-SD participants reported a higher rate of moderate and severe IBS-related gastrointestinal symptoms in the current study. Contrarily, IBS-NM participants experienced more mild symptoms with 3.5% of IBS-NM participants being in remission. Instead, IBS-SD participants had a lower QOL perspective, scoring lower scores in all IBS-QOL domains except for the sexual domain than those in the IBS-NM group. Baseline levels of cortisol and serotonin did not significantly differ between those with subthreshold depression and with normal mood.

**Table 1:** Sociodemographic and anthropometric data in overall IBS participants (n = 110).

Characteristics	Results
Sociodemographic	
Age median (IQR)	26 (12)
Gender, n (%)	
Male	22 (20.0)
Female	88 (80.0)
Race, n (%)	
Malay	91 (82.7)
Chinese	5 (4.6)
Indian	3 (2.7)
Others	11 (10.0)
Marital status, n (%)	
Single	64 (58.2)
Married	43 (39.1)
Divorced	3 (2.7)
Education level, n (%)	
Primary	2 (1.8)
Secondary	23 (20.9)
Diploma	18 (16.4)
Undergraduate	60 (54.6)
Postgraduate	7 (6.4)
Healthcare worker, n (%)	
Yes	23 (20.9)
No	87 (79.1)
IBS subtypes, n (%)	
IBS-C	44 (40.0)
IBS-D	33 (30.0)
IBS-M	22 (20.0)
IBS-U	11 (10.0)
Anthropometric	
BMI categories, n (%)	
Underweight (< 18.5 kg/m^2^)	6 (5.5)
Normal (18.5–24.9 kg/m^2^)	50 (45.5)
Pre-obese (25.0–29.9 kg/m^2^)	30 (27.3)
Obese (> 30.0 kg/m^2^)	24 (21.8)
Waist circumference (mean ± SD cm)	79.1 ± 10.1
Central obesity, n (%)	
Yes	47 (42.7)
No	63 (57.3)

Data expressed in mean ± standard deviation, median (interquartile range) or in percentage (%) based on data distribution.

*IQR* interquartile range, *SD* standard deviation.

Table 2 summarizes the parameter differences between all four intervention groups. Significant differences were noted in the depressive symptoms scores (p < 0.001) and total IBS-QOL scores (p = 0.009), particularly dysphoria (p = 0.0013), body image (p = 0.018), health worry (p = 0.028) food avoidance (p = 0.031), social reaction (p = 0.042), and relationship (p = 0.033). The only parameter that indicated a significant difference at baseline was the serum cortisol (p = 0.033) across groups. There was no significant difference in all the other parameter at baseline.

**Table 2:** Comparison of baseline measures between all groups (n = 110).

Variables	NM-placebo (n = 29)	NM-probiotic (n = 28)	SD-placebo (n = 27)	SD-probiotic (n = 26)	p-value
Depression symptoms score (mean ± SD)					
CESD-R score	5.6 ± 4.3	5.4 ± 5.2	22.1 ± 6.7	23.8 ± 8.7	0.000**
Quality of life (median, IQR)					
Total IBS-QOL score	80.9 (70.6, 91.5)	86.8 (77.0, 94.9)	66.9 (50.0, 85.3)	77.6 (64.3, 89.7)	0.009*
Domain 1: dysforia	84.4 (78.1, 93.8)	90.6 (81.3, 96.9)	65.6 (43.8, 90.6)	84.4 (68.8, 93.8)	0.013*
Domain 2: interference with activity	82.1 (58.9, 92.9)	83.9 (73.2, 92.9)	67.9 (42.9, 89.3)	75.0 (57.1, 90.2)	0.117
Domain 3: body image	81.3 (68.8, 96.9)	87.5 (76.6, 93.8)	62.5 (43.8, 87.5)	75.0 (62.5, 93.8)	0.018*
Domain 4: health worry	75.0 (66.7, 91.7)	83.3 (52.1, 91.7)	50.0 (33.3, 75.0)	70.8 (47.9, 91.7)	0.028*
Domain 5: Food avoidance	58.3 (58.3, 83.3)	83.3 (66.7, 97.9)	58.3 (33.3, 83.3)	66.7 (50.0, 83.3)	0.031*
Domain 6: social reaction	81.3 (65.6, 96.9)	87.5 (76.6, 100.0)	62.5 (50.0, 93.8)	81.3 (60.9, 93.8)	0.042*
Domain 7: relationship	91.7 (75.0, 100.0)	91.7 (83.3, 100.0)	83.3 (58.3, 100.0)	83.3 (64.6, 91.7)	0.022*
Domain 8: sexual	100.0 (87.5, 100.0)	100.0 (100.0, 100.0)	100.0 (75.0, 100.0)	100.0 (87.5, 100.0)	0.091
Hormone serum level (ng/ml)(mean ± SD)					
Cortisol	176.5 ± 148.3	352.6 ± 235.9	189.0 ± 86.9	341.9 ± 310.7	0.033*
Serotonin (min log)	1.9 ± 0.3	2.2 ± 0.5	2.0 ± 0.4	2.1 ± 0.3	0.101
IBS symptoms severity (mean ± SD)					
Total IBS-SSS score	205.5 ± 68.5	203.0 ± 81.3	235.9 ± 70.3	236.7 ± 93.1	0.212
Subscale 1: abdominal pain severity	43.8 ± 19.5	40.7 ± 23.6	49.3 ± 20.4	49.2 ± 24.0	0.387
Subscale 2: number of days with abdomina pain	36.4 ± 24.8	41.3 ± 30.1	43.3 ± 29.9	45.6 ± 30.1	0.670
Subscale 3: abdominal distension	40.9 ± 20.5	35.7 ± 19.3	35.0 ± 19.8	42.7 ± 21.1	0.417
Subscale 4: bowel habit dissatisfaction	43.1 ± 19.4	45.7 ± 20.3	57.2 ± 20.7	51.5 ± 22.9	0.061
Subscale 5: life disruption	41.4 ± 18.3	40.0 ± 22.4	51.1 ± 20.3	47.7 ± 24.7	0.183
IBS severity category, *n* (%)					
Remission (< 75)	–	2 (7.1)	–	–	0.258
Mild (75–174)	8 (27.6)	8 (28.6)	5 (18.5)	4 (15.4)	
Moderate (175–299)	18 (62.1)	14 (50.0)	16 (59.3)	17 (65.4)	
Severe (≥ 300)	3 (10.3)	4 (14.3)	6 (22.2)	5 (19.2)	

Data are expressed as means ± standard deviations, medians (interquartile range) or percentages (%) based on data distribution. Data was analyzed with one-way ANOVA and Kruskal–Wallis analyses according to data distribution (*p < 0.05, **p < 0.001).

*ANOVA* analysis of variance, *IQR* interquartile range, *SD* standard deviation, *NM* normal mood, *SD* subthreshold depression.

### Primary outcomes

**Figure 3:**
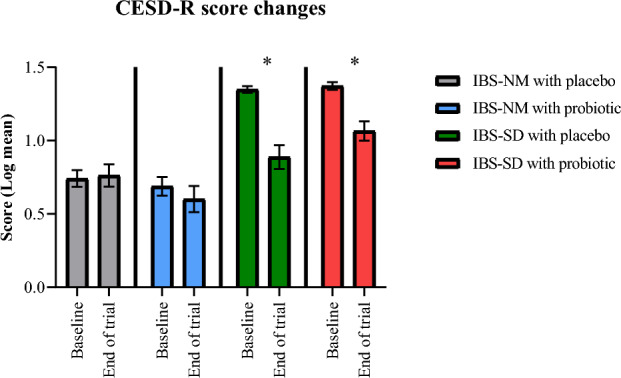
The bar chart shows the changes in CESD-R scores of each group following the intervention with cultured milk drinks. The data were calculated using the paired t-test with data presented as mean and standard error of mean (*p < 0.05).

The CESD-R scores in IBS-SD participants with probiotic showed significant reduction with log mean difference of 0.31 (95% CI 0.19, 0.43; p < 0.001; Fig. 3; Supplementary Table 2S). A significant reduction in CESD-R log mean scores was also noted among participants receiving placebo with log mean difference of 0.46 (95% CI 0.29, 0.63; p < 0.001; Fig. 3; Supplementary Table 2S). The CESD-R score increased slightly in IBS-NM participants who received either placebo or *lactobacillus*-containing cultured milk drinks, but no significant changes were noted (Fig. 3). The depression score changes between groups were statistically significant with effect size of 0.434 (Mean difference 0.21; 95% CI 0.13, 0.28; p < 0.001; Supplementary Table 8S) using the GLM ANOVA repeated measures analysis. Similarly, a significant reduction in depression symptoms score were reported after covariate adjustment with ANCOVA (Mean difference 0.20; 95% CI 0.12, 0.28; p < 0.001; effect size 0.304; Supplementary Table 8S). A post hoc analysis was performed to further identify the differences between individual groups using Bonferroni method. Significant differences were noted between IBS-NM placebo with IBS-SD placebo (mean difference − 0.366, p < 0.001, 95% CI − 0.566, − 0.165; Supplementary Table 9S) and IBS-SD probiotic (mean difference − 0.467, p < 0.001, 95% CI − 0.67, − 0.265; Supplementary Table 9S). Despite showing statistically significant differences between IBS-NM probiotic with IBS-SD placebo (mean difference − 0.473, p < 0.001, 95% CI − 0.675, − 0.270; Supplementary Table 9S) and IBS-SD probiotic (mean difference − 0.574, p < 0.001, 95% CI − 0.779, − 0.370; Supplementary Table 9S), no significant difference was noted between IBS-SD placebo and IBS-SD probiotic groups. Both IBS-SD patients receiving probiotic and placebo experienced a decline in CESD-R scores resulting in more participants with CESD-R score of less than 16. Consequently, a rise of newly reported normal mood category (CESD-R < 16) was recorded (Supplementary Fig. 1S). At post-intervention, 65.4% and 74.1% of IBS-SD participants who received probiotic and placebo, respectively, were categorized as having normal mood (Supplementary Table 3S). In contrast, a newly reported subthreshold depression category (CESD-R > 16) was observed among IBS-NM participants after completion of the intervention comprising probiotic (10.7%) and placebo (6.9%) (Supplementary Table 3S).

### Secondary outcomes

**Figure 4:**
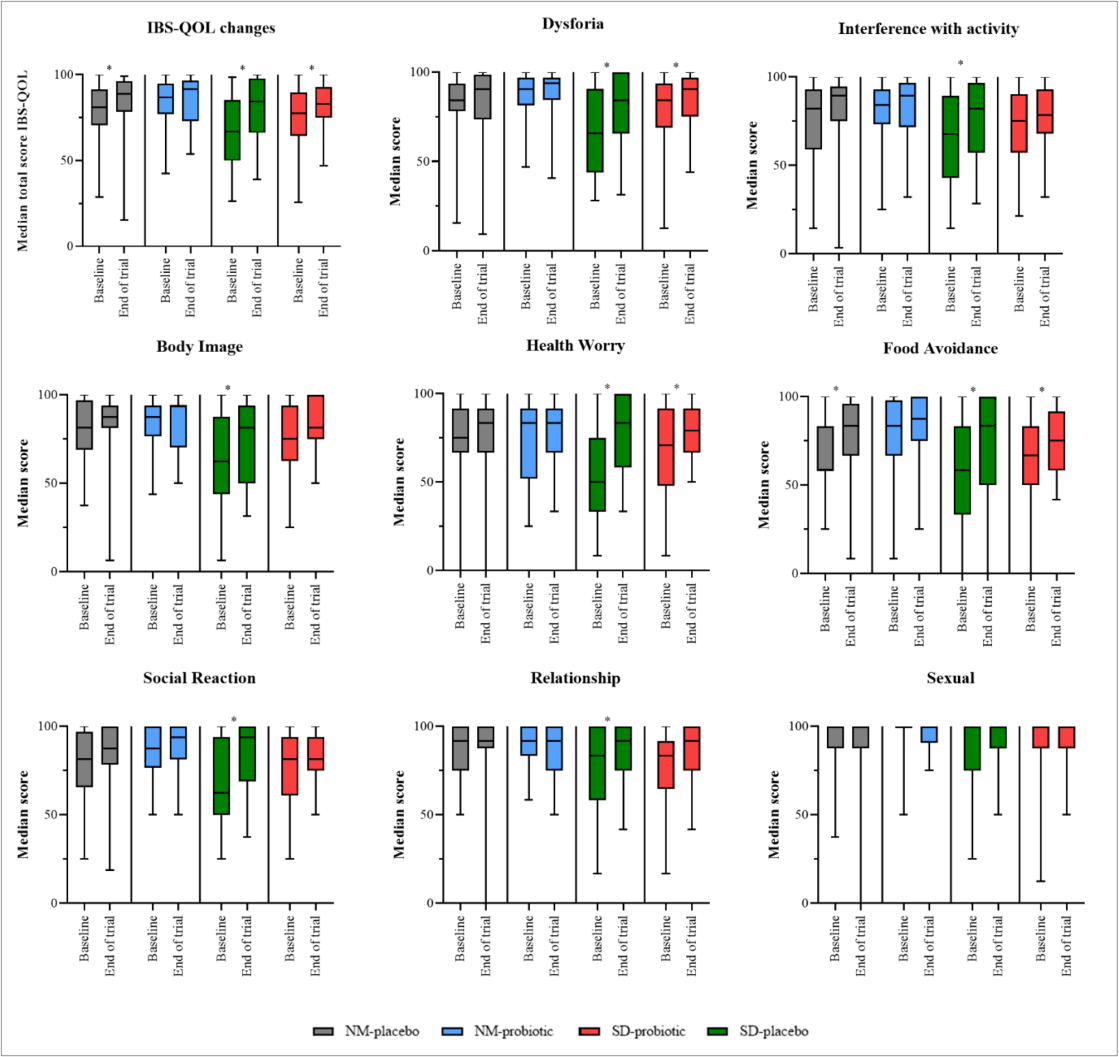
The box plot shows the changes in total and eight domains of IBS-QOL scores after the cultured milk drink intervention for each group. *p*-values were obtained using the median and interquartile range of the data and the non-parametric Wilcoxon Signed-Rank test (***pe < 0.05).

After probiotic intervention, the total IBS-QOL scores improved by 7.1%, from 77.6 to 83.1 for IBS-SD participants (Fig. 4; Supplementary Table 4S). Similar outcomes were observed in both IBS-SD and IBS-NM receiving placebo. Significant improvements of 26.4% (66.9–84.6) and 10% (80.9–89.0) were reported in respective groups. However, no significant difference was observed in the IBS-NM participants consuming probiotic drink. Three IBS-QOL domains for IBS-SD with probiotic drink improved significant: dysphoria (p = 0.039), health worry (p = 0.018), and food avoidance (p = 0.04). Concurrently, the IBS-SD participants given a placebo exhibited significant improvements in almost all except the sexual domain (Fig. 4; Supplementary Table 4S). Only one domain of IBS-QOL (food avoidance; p = 0.01) was significantly improved in IBS-NM participants treated with placebo. Despite an increase in all IBS-QOL domain scores, the changes for IBS-NM participants receiving probiotic were minimal and insignificant. Analysis between group using the Kruskal–Wallis method showed that the differences in overall IBS-QOL score (p = 0.044; Supplementary Table 4S), health worry domain (p = 0.007; Supplementary Table 4S) and sexual domain (p = 0.045; Supplementary Table 4S) were significant. A post hoc analysis using Mann–Whitney U-test showed significant difference in overall IBS-QOL scores between IBS-NM placebo with IBS-SD placebo (Z = − 1.959, U = 272, p = 0.0049; Supplementary Table 10S) and IBS-NM probiotic with IBS-SD placebo (Z = − 2.576, U = 225, p = 0.01; Supplementary Table 10S). Similarly, health worry domain was significantly differ between IBS-NM placebo with IBS-SD placebo (Z = − 3.108, U = 204, p = 0.002; Supplementary Table 10S) and IBS-NM probiotic with IBS-SD placebo (Z = − 2.887, U = 208.5, p = 0.004; Supplementary Table 10S). Meanwhile, only IBS-NM probiotic with IBS-SD placebo showed significant difference in sexual domain (Z = − 2.760, U = 217.5, p = 0.006; Supplementary Table 10S). No significant difference was noted between IBS-SD placebo and IBS-SD probiotic.

**Figure 5:**
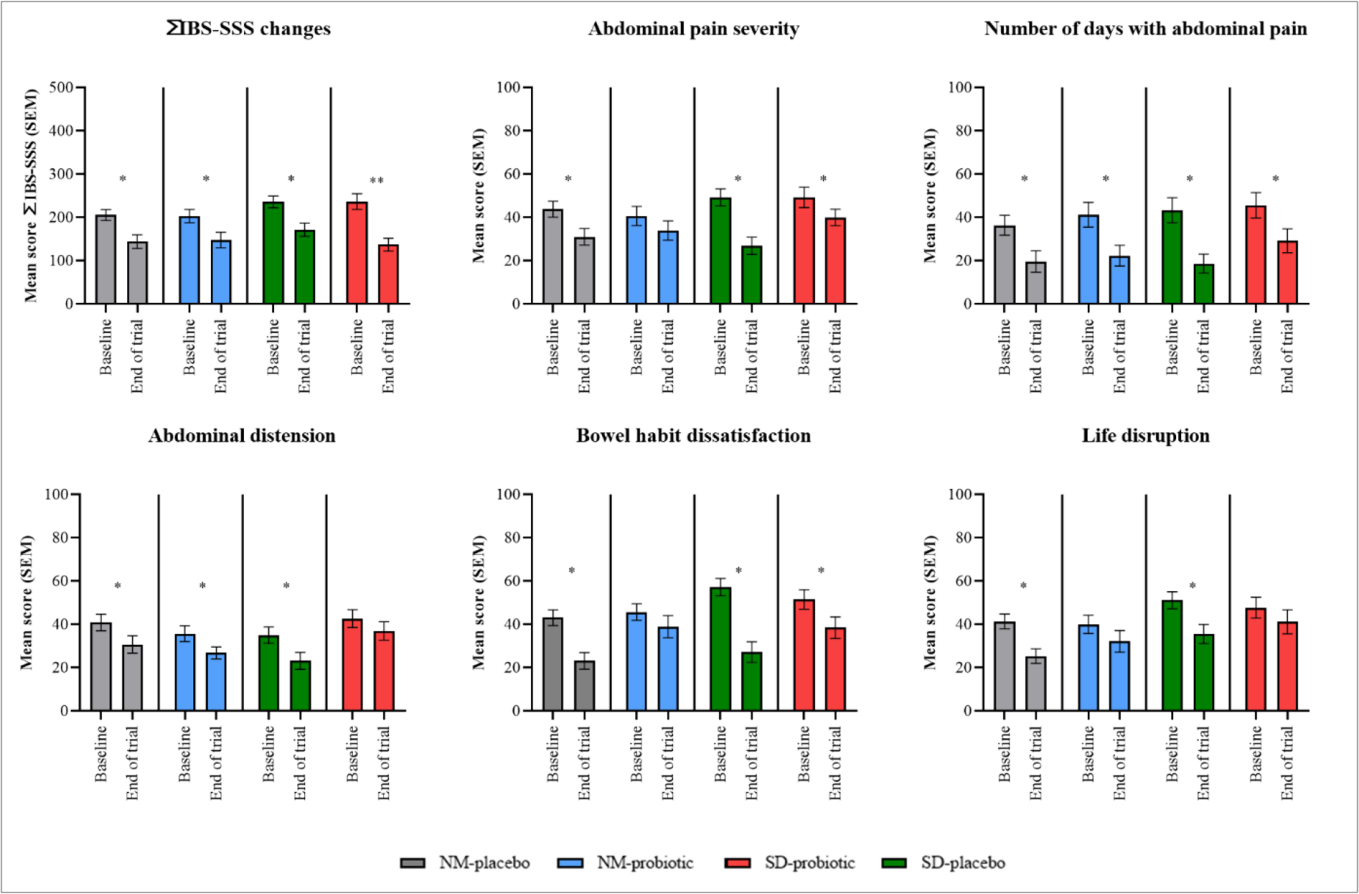
The box plot shows the changes overall (∑IBS − SSS) and five domains in IBS-SSS scores after the intervention with cultured milk drink for each group. The symbol * and ** represent the *p*-value comparing pre- and post-intervention outcomes. Data provided as mean and standard error of mean (SEM) (*p < 0.05, **p < 0.001).

IBS-SD treated with probiotic showed significant gastrointestinal symptoms improvement with a 42.1% drop in score (from 236.73 to 93.11; p < 0.001; Supplementary Table 5S). However, analysis with GLM ANOVA revealed no significant difference between all four groups (mean difference 69.58; 95% CI 56.71, 82.44; p = 0.170; Supplementary Table 8S). The ANCOVA analysis showed a significant difference between group with covariate adjustments for total IBS-SSS scores (Supplementary Table 8S). Three symptoms in the questionnaire (abdominal pain severity, number of days with stomach pain, and bowel habit dissatisfaction; Fig. 5) showed a substantial improvement. A post hoc analysis with covariate (gender) adjustment was performed which revealed significant difference between IBS-NM probiotic with IBS-SD placebo in overall IBS-SSS scores (mean difference − 53.428; 95% CI − 103.15, − 3.697; p = 0.028; Supplementary Table 11S), abdominal pain severity (mean difference − 15.057; 95% CI − 29.934, − 0.167; p = 0.046; Supplementary Table 11S) and bowel habit dissatisfaction (mean difference − 23.186; 95% CI − 40.846, − 5.525; p = 0.004; Supplementary Table 11S). Similarly, the overall IBS-SSS score was statistically significant between IBS-SD placebo and IBS-SD probiotic groups (mean difference − 53.981; 95% CI − 104.278, − 3.685; p = 0.028; Supplementary Table 11S). While IBS-SD treated with a placebo demonstrated significant improvement in all symptoms, with the overall IBS-SSS score declined from 235.93 to 171.48 (Supplementary Table 5S). The IBS-NM participants who received a placebo experienced a rapid improvement in their overall questionnaire score across all five symptoms (Fig. 5; Supplementary Table 5S). However, only two symptoms (abdominal pain and abdominal distension) showed significant improvements in IBS-NM participants who received probiotic. Surprisingly, neither IBS individual who received probiotics showed substantial changes in the life disruption domain (Fig. 5).

The IBS severity pattern shifted from higher rate of moderate-severe levels towards higher rate of remission-mild symptoms across all groups at post-intervention, irrespective of placebo or probiotic-supplemented (Supplementary Fig. 2S). The IBS-SD participants with probiotic showed increased remission and mild IBS reports by 15.4% and 23.1%, consecutively (Supplementary Table 6S). Likewise, the IBS-SD with placebo showed 59.3% raise in remission-mild IBS with reduction in moderate-severe symptoms (Supplementary Table 6S). Both IBS-NM participants showed increment in the remission-mild IBS at post-intervention by 48.3% and 28.6% in respective groups (Supplementary Fig. 2S).

**Figure 6:**
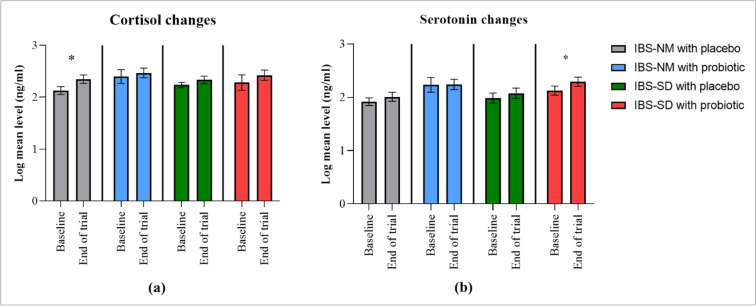
The bar chart shows the changes in hormone levels post cultured milk drink intervention: (**a**) cortisol hormone; (**b**) serotonin hormone. *p*-value obtained from the paired t-test and data expressed in mean and standard error of mean (*p < 0.05).

Serotonin serum levels among IBS-SD participants with probiotic increased significantly with mean difference of 0.166 (95% CI 0.255, − 0.077; p = 0.002; effect size 1.128; Fig. 6; Supplementary Table 7S). The IBS-NM participants who received probiotic or a placebo did not exhibit any significant changes in their serum serotonin levels. The cortisol serum level in IBS-SD participants did not significantly change despite an increasing pattern following probiotic intervention. Only IBS-NM participants who were given a placebo had a cortisol serum level significantly increased with log mean difference of 0.219 (95% CI − 0.374, − 0.063; p = 0.008; effect size 0.679; Fig. 6; Supplementary Table 7S). All other groups did not show any significant changes. However, even after controlling for covariate, no significant differences in serum cortisol and serotonin were observed between groups (Supplementary Table 8S).

### Adverse effects (AE)

The rate of AE was 18.2% (n = 20): placebo, 9 and probiotic, 11. 90% with AE experienced digestive-related symptoms, while 10% had side effects unrelated to digestion. Side effects include hunger with early satiety, nausea, vomiting, loose stool, stool incontinence, bloating and more severe abdominal pain. Throughout the study, six participants had undergone quarantine due to close contact or positive Covid-19 test, either at home or in a quarantine centre (n = 2).

## Discussion

In our previous study, one third of IBS patients experienced symptoms of subthreshold depression which undoubtedly aiding the progression of major depressive episode without early intervention in place. This study was designed to better understand how cultured milk drinks containing dual probiotic strains can reduce depression risk among IBS patients. The inevitable growth in the frequency of depression worldwide and its incapacitating character make it imperative to take psychological issues into account while treating IBS patients^32^. Despite its positive outcomes as psychobiotics, there is limited evidence of their ability to help IBS patients with psychological issues, specifically depression.

In this study, two bottles of cultured milk drink containing 1 billion cfu of dual strain *lactobacillus* for 12-week were able to ameliorate depression in IBS patients with subthreshold depression, as seen in previous trials^26,27^. Additionally, IBS-SD participants who received a placebo in our study also displayed a decrease in their depression symptoms. This finding implied that additional features such as positive attitude and perspective towards the interventional products received, may contribute to the treatment efficacy^27,33^. However, earlier studies using either capsulated or dairy products containing lactic acid bacteria demonstrated no significant changes in depression scores among IBS patients^34^. Similarly, supplementation with multispecies probiotics has not been shown to be superior in alleviating the symptoms of depression^35^.

Prior study suggested that QOL perspective influences patients’ anticipated risk of depression, with healthy persons without gastrointestinal symptoms having lower depression scores than untreated IBS patients^36^. Unlike previous study where probiotics showed superiority in improving all IBS-QOL domains, our study showed some improvement only in several QOL domains^23^. IBS-SD participants who received probiotics in our study benefitted significantly, particularly in three domains relatable to behaviour and psychological phenotypes. This improvement may contribute to the reduction in depressive symptoms. The IBS-SD participants in our study who received a placebo had a corresponding outcome with significant improvement in all domains of QOL measures except the sexual domain, due to predominance of single and unmarried participants. The findings were consistent with a prior trial of a 4-week placebo programme that successfully improved dysphoria and health worry aspects in IBS patients^23^. We hypothesize that the improvement in perceived QOL measures among our IBS patients was caused by the restoration of gut microbiota diversity from *lactobacillus* supplementation^37^. We hypothesise that the probiotic cultured milk drink's ability to restore gut microbiota helped our IBS participants' gastrointestinal symptoms, even though this was not explored in our study.

In this study, both probiotics and placebo reduced the severity of IBS symptoms, as seen in a prior clinical trial^37^. However, IBS-SD treated with probiotic only effectively reduced the frequency and severity of abdominal pain while improving bowel habit satisfaction. This demonstrated that the efficacy of psychobiotic intervention is dependent on other factors, such as the IBS subtypes, where treatment efficacy varies^38^. For instance, a 30-day *lactobacillus*-containing cultured milk drink intervention decreased intestinal transit time, alleviating the constipation symptom experienced by IBS-C patients^20^. Other studies found that either probiotics or placebo relieved gastrointestinal symptoms without any significant differences between IBS subtypes^39,40^. However, a prior trial of a dual strain *lactobacillus* intervention failed to demonstrate its effectiveness in relieving IBS symptoms such as abdominal pain^41^. These diversities nullified the superiority of probiotics in alleviating the gastrointestinal symptoms associated with IBS.

Cortisol level was significantly elevated among IBS-NM participants who consumed a placebo cultured milk drink. Cortisol, which is frequently increased in individuals with IBS with chronic stress, regulates the immune system via the HPA axis^9^. The rise in cortisol levels may be linked to visceral hypersensitivity, which reflects their gastrointestinal complaints^42^. Our current findings were consistent with a recent trial that demonstrated no significant reduction in cortisol levels after 4-week of probiotic consumption^43^. In contrast, a prior study that used a mix of *lactobacillus* and *bifidobacterium* strains for 30 days was successful in lowering the urine cortisol level^44^. In recent years, researchers proposed a theory of hypocortisolism in which they predicted lower cortisol levels in IBS adults under chronic stress conditions^45^. It was hypothesized that the HPA axis hyperactivation persisted despite the reduced cortisol synthesis, leading to increased CRH receptor activation in the brain and digestive system. Consequently, serotonin secretion increases with time, resulting in impaired gastrointestinal motility and causing hyperalgesia. A 12-week probiotic treatment was able to lower cortisol expression, strongly suggesting the significance of probiotics in regulating cortisol production, which reduces the severity and duration of gastrointestinal symptoms such as abdominal pain^24^.

A possible explanation for the improvement in depressive and gastrointestinal symptoms reported in this study could be the rise in serum serotonin level among IBS-SD participants who consumed the *lactobacillus*-containing cultured milk drink. It has been previously discovered that serotonin dysregulation leads to immune system dysregulation, which in turn causes depressive symptoms among IBS patients^46,47^. The significant rise reflects increased free circulating serotonin molecules in the body, which are commonly transported using protein carriers that express serotonin-transporter (SERT) receptors^48^. Despite being essential for gut-brain transmission, only two percent of serotonin generated in the intestines enters the bloodstream. Serotonin reuptake into enterocytes via SERT receptors may assist to regulate gut motility, mucus secretion, and bowel habit, potentially enhanced with probiotic use^48^. Despite the increasing trend from baseline reading, no significant changes in serotonin levels were found among IBS participants after the multispecies probiotic intervention^27,41^. However, we acknowledged that serotonin expression differs between IBS subtypes such that higher SERT expression in IBS-C patients decreasing the intestinal serotonin bioavailability^48^. Hence, to avoid unintended consequences, experts' recommendations and guidance on the probiotic’s choice are justified.

Despite the favorable change in serotonin levels caused by probiotics, the superiority of probiotics cannot be determined merely by this study. We acknowledge that placebo cultured milk drink showed good evidence in alleviating the other subjective measures in our study including its influence over depression symptoms. The use of self-reported questionnaires was vulnerable to the subjective perceptions of an individual which may have been influenced by their psychological belief and viewpoint towards the intervention received^27,33^. Several factors, including the symbiotic interaction between the host and gut microbiota, may contribute to the efficacy of probiotic interventions^49^. In our study, the efficacy of the dual strain *lactobacillus* was influenced by the choice of probiotic carrier and the features of our recruited participants. The use of pasteurised milk as the primary probiotic carrier may improve potency in reaching the proximal colon for optimal function where it has clearly improved IBS symptoms and QOL but not the depression risk score^50^. Furthermore, due to the nature of the condition, IBS-C patients may recognise changes in their stool quality and bowel habit frequency better than other subtypes.

Gender, perceived stress level, and educational level are additional factors that may have contributed to our findings. The gut microbiota plays a role in an individual’s risk of psychological difficulties during challenging circumstances, and is influenced by gender, with women showing higher cortisol levels than men^9^. Therefore, a significant difference between male and female participants in our study may affect the baseline cortisol level which consecutively influence the study outcomes, contributed by their biological responses towards the cultured milk drink treatment. Majority of our study participants were university students, who may have academic-related stressors resulting in disrupted cortisol production and gut function^9^. The Covid-19 pandemic outbreak during the study phase, is a recognized crucial element that has the potential to exacerbate the current depression or precipitate fresh episodes of symptoms^51^. Prolonged stress and anxiety attacks were more apparent during the pandemic, resulting in the recurrence of gastrointestinal symptoms^51,52^. Six participants in our study were placed in quarantine due to Covid-19 related event, putting them at risk for psychological threat throughout the trial period. Therefore, the cortisol baseline levels and changes shown in this study may not be representative of the actual population.

Unmeasured dietary intake is an essential element that may influence our findings, including the use of prebiotics, dietary fibres and implementation of low FODMAP diet^53–55^*.* These dietary elements enhanced and enriched the function of *lactobacillus acidophilus* in the colon, which symbiotically modifies gut microbial diversity, and reduces IBS symptoms. Moreover, the cultured milk drink used in this study contained fructose and polydextrose which are examples of FODMAP that potentially could influence the gut motility. Despite growing evidence that probiotics can effectively treat depressive symptoms in IBS patients, this treatment is currently not considered as an adjuvant preventive therapy due to inconsistent outcomes across the world. However, its benefit as an adjunct therapy to alleviate depression symptoms among IBS patients is suggestive.

## Conclusion

The *lactobacillus*-containing cultured milk drink showed potential anti-depressive properties among IBS participants who are at risk of developing depression. Its efficacy in ameliorating depressed symptoms was suggested by a possible regulatory mechanism of serotonin hormone involved in regulation of mood through gut microbiota symbiosis. However, the use of probiotic cultured milk drink as a sole therapy for depression symptoms is still unjustified due to various confounding factors including dietary intake, physical activity, and baseline gut microbiome taxonomy, which may be explored further in future study. Future studies with a larger sample size from other tertiary centers and a clear description of study population may assist to determine the probiotics’ true preventative benefit in treating depression.

### Supplementary Information

Supplementary Table 1.

Supplementary Table 2.

Supplementary Table 3.

Supplementary Table 4.

Supplementary Table 5.

Supplementary Table 6.

Supplementary Table 7.

Supplementary Table 8.

Supplementary Table 9.

Supplementary Table 10.

Supplementary Table 11.

Supplementary Figure 1.

Supplementary Figure 2.

Supplementary Information 14.

## Data Availability

All data generated or analysed during this study are included in this published article and its supplementary information files.

## References

[1] Riddle MS, Welsh M, Porter CK, Nieh C, Boyko EJ, Gackstetter G (2016). The epidemiology of irritable bowel syndrome in the US military: Findings from the Millennium Cohort Study. Am. J. Gastroenterol..

[2] Icenhour A, Witt ST, Elsenbruch S, Lowén M, Engström M, Tillisch K (2017). Brain functional connectivity is associated with visceral sensitivity in women with irritable bowel syndrome. NeuroImage Clin..

[3] Chumpitazi BP, Self MM, Czyzewski DI, Cejka S, Swank PR, Shulman RJ (2016). Bristol Stool Form Scale reliability and agreement decreases when determining Rome III stool form designations. Neurogastroenterol. Motil..

[4] Oka P, Parr H, Barberio B, Black CJ, Savarino EV, Ford AC (2020). Global prevalence of irritable bowel syndrome according to Rome III or IV criteria: A systematic review and meta-analysis. Lancet Gastroenterol. Hepatol..

[5] Rahman MM, Mahadeva S, Ghoshal UC (2017). Epidemiological and clinical perspectives on irritable bowel syndrome in India, Bangladesh and Malaysia: A review. World J. Gastroenterol..

[6] Gwee KA, Gonlachanvit S, Ghoshal UC, Chua AS, Miwa H, Wu J (2019). Second Asian consensus on irritable bowel syndrome. J. Neurogastroenterol. Motil..

[7] Zhang W, Zhao D, Qi Q, Long X, Li Y, Wang P (2018). Nr2b-containing nmda receptors contribute to diarrhea-predominant irritable bowel syndrome. Oncotarget.

[8] Lee C, Doo E, Choi JM, Jang SH, Ryu HS, Lee JY (2017). The increased level of depression and anxiety in irritable bowel syndrome patients compared with healthy controls: Systematic review and meta-analysis. J. Neurogastroenterol. Motil..

[9] Batabyal A, Bhattacharya A, Thaker M, Mukherjee S (2021). A longitudinal study of perceived stress and cortisol responses in an undergraduate student population from India. PLoS ONE.

[10] Mokhtar NM, Bahrudin MF, Abd Ghani N, Abdul Rani R, Raja Ali RA (2020). Prevalence of subthreshold depression among constipation-predominant irritable bowel syndrome patients. Front. Psychol..

[11] Stasi C, Nisita C, Cortopassi S, Corretti G, Gambaccini D, De Bortoli N (2017). Subthreshold psychiatric psychopathology in functional gastrointestinal disorders: can it be the bridge between gastroenterology and psychiatry?. Gastroenterol. Res. Pract..

[12] Zhang R, Peng X, Song X, Long J, Wang C, Zhang C (2022). The prevalence and risk of developing major depression among individuals with subthreshold depression in the general population. Psychol. Med..

[13] Jeuring HW, Comijs HC, Deeg DJ, Stek ML, Huisman M, Beekman AT (2018). Secular trends in the prevalence of major and subthreshold depression among 55–64-year olds over 20 years. Psychol. Med..

[14] Padhi P, Worth C, Zenitsky G, Jin H, Sambamurti K, Anantharam V (2022). Mechanistic insights into gut microbiome dysbiosis-mediated neuroimmune dysregulation and protein misfolding and clearance in the pathogenesis of chronic neurodegenerative disorders. Front. Neurosci..

[15] Hill C, Guarner F, Reid G, Gibson GR, Merenstein DJ, Pot B (2014). The International Scientific Association for Probiotics and Prebiotics consensus statement on the scope and appropriate use of the term probiotic. Nat. Rev. Gastroenterol. Hepatol..

[16] Aizawa E, Tsuji H, Asahara T, Takahashi T, Teraishi T, Yoshida S (2016). Possible association of Bifidobacterium and Lactobacillus in the gut microbiota of patients with major depressive disorder. J. Affect. Disord..

[17] Bennet SM, Palsson O, Whitehead WE, Barrow DA, Törnblom H, Öhman L (2018). Systemic cytokines are elevated in a subset of patients with irritable bowel syndrome but largely unrelated to symptom characteristics. Neurogastroenterol. Motil..

[18] Xie R, Jiang P, Lin LI, Jiang J, Yu B, Rao J (2020). Oral treatment with Lactobacillus reuteri attenuates depressive-like behaviors and serotonin metabolism alterations induced by chronic social defeat stress. J. Psychiatr. Res..

[19] Zou W, Feng R, Yang Y (2018). Changes in the serum levels of inflammatory cytokines in antidepressant drug-naïve patients with major depression. PLoS ONE.

[20] Mokhtar N, Jaafar N, Alfian E, Rathi NM, Rani RA, Ali RR (2021). Clinical assessment and cytokines level in constipation-predominant irritable bowel syndrome participants treated with Lactobacillus-containing cultured milk drink. Acta Gastro Enterol. Belg..

[21] Zhang L, Liu YX, Wang Z, Wang XQ, Zhang JJ, Jiang RH (2019). Clinical characteristic and fecal microbiota responses to probiotic or antidepressant in patients with diarrhea-predominant irritable bowel syndrome with depression comorbidity: A pilot study. Chin. Med. J..

[22] Rudzki L, Ostrowska L, Pawlak D, Małus A, Pawlak K, Waszkiewicz N, Szulc A (2019). Probiotic Lactobacillus Plantarum 299v decreases kynurenine concentration and improves cognitive functions in patients with major depression: A double-blind, randomized, placebo controlled study. Psychoneuroendocrinology.

[23] Ishaque SM, Khosruzzaman SM, Ahmed DS, Sah MP (2018). A randomized placebo-controlled clinical trial of a multi-strain probiotic formulation (Bio-Kult®) in the management of diarrhea-predominant irritable bowel syndrome. BMC Gastroenterol..

[24] Lalitsuradej, E., Sirilun, S., Chaiyasut, C., & Sittiprapaporn, P. A preliminary study on effect of lactobacillus paracasei hii01 on cortisol in fatigue subjects. In *2019 16th International Conference on Electrical Engineering/Electronics, Computer, Telecommunications and Information Technology (ECTI-CON) 2019 Jul 10* (pp. 466–469). IEEE.

[25] Ma T, Jin H, Kwok LY, Sun Z, Liong MT, Zhang H (2021). Probiotic consumption relieved human stress and anxiety symptoms possibly via modulating the neuroactive potential of the gut microbiota. Neurobiol. Stress..

[26] Majeed M, Nagabhushanam K, Arumugam S, Majeed S, Ali F (2018). Bacillus coagulans MTCC 5856 for the management of major depression with irritable bowel syndrome: A randomised, double-blind, placebo controlled, multi-centre, pilot clinical study. Food Nutr. Res..

[27] Pinto-Sanchez MI, Hall GB, Ghajar K, Nardelli A, Bolino C, Lau JT (2017). Probiotic Bifidobacterium longum NCC3001 reduces depression scores and alters brain activity: A pilot study in patients with irritable bowel syndrome. Gastroenterology..

[28] Francis CY, Morris J, Whorwell PJ (1997). The irritable bowel severity scoring system: a simple method of monitoring irritable bowel syndrome and its progress. Aliment. Pharmacol. Ther..

[29] Van Dam NT, Earleywine M (2011). Validation of the Center for Epidemiologic Studies Depression Scale—Revised (CESD-R): Pragmatic depression assessment in the general population. Psychiatry Res..

[30] Patrick DL, Drossman DA, Frederick IO, Dicesare J, Puder KL (1998). Quality of life in persons with irritable bowel syndrome (development and validation of a new measure). Digest. Dis. Sci..

[31] Drossman DA, Chang L, Bellamy N, Gallo-Torres HE, Lembo A, Mearin F (2011). Severity in irritable bowel syndrome: A Rome Foundation Working Team report. Off. J. Am. Coll. Gastroenterol..

[32] Depression WH (2017). Other common mental disorders: global health estimates.

[33] Laterza L, Napoli M, Petito V, Scaldaferri F, Gaetani E, Gasbarrini A (2021). Evaluation of tolerability and major factors affecting the adherence to probiotic therapy in patients with irritable bowel syndrome: A prospective, observational, real-life study. Minerva Med..

[34] Schaub AC, Schneider E, Vazquez-Castellanos JF, Schweinfurth N, Kettelhack C, Doll JP (2022). Clinical, gut microbial and neural effects of a probiotic add-on therapy in depressed patients: A randomized controlled trial. Transl. Psychiatry..

[35] Akkasheh G, Kashani-Poor Z, Tajabadi-Ebrahimi M, Jafari P, Akbari H, Taghizadeh M (2016). Clinical and metabolic response to probiotic administration in patients with major depressive disorder: A randomized, double-blind, placebo-controlled trial. Nutrition..

[36] Michalsen VL, Vandvik PO, Farup PG (2015). Predictors of health-related quality of life in patients with irritable bowel syndrome: A cross-sectional study in Norway. Health Qual. Life Outcomes..

[37] Alexandru BA, Rat LA, Moldovan AF, Mihancea P, Mariș L (2022). An open-label trial study of quality-of-life assessment in irritable bowel syndrome and their treatment. Medicina..

[38] Liu Y, Yu X, Yu L, Tian F, Zhao J, Zhang H (2021). Lactobacillus plantarum CCFM8610 alleviates irritable bowel syndrome and prevents gut microbiota dysbiosis: A randomized, double-blind, placebo-controlled, pilot clinical trial. Engineering..

[39] Zhang X, Chen S, Zhang M, Ren F, Ren Y, Li Y (2021). Effects of fermented milk containing Lacticaseibacillus paracasei strain Shirota on constipation in patients with depression: A randomized, double-blind, placebo-controlled trial. Nutrients..

[40] Dapoigny M, Piche T, Ducrotte P, Lunaud B, Cardot JM, Bernalier-Donadille A (2012). Efficacy and safety profile of LCR35 complete freeze-dried culture in irritable bowel syndrome: A randomized, double-blind study. World J. Gastroenterol. WJG..

[41] Maixent JM, Pons O, Sennoune SR, Sadrin S (2020). Clinical effects of Lactobacillus strains as probiotics in the treatment of irritable bowel syndrome: Results from the LAPIBSS trial: Future objectives. Cell. Mol. Biol..

[42] Ogłodek EA (2022). Changes in the serum concentration levels of serotonin, tryptophan and cortisol among stress-resilient and stress-susceptible individuals after experiencing traumatic stress. Int. J. Environ. Res. Public Health..

[43] Rode J, Edebol Carlman HM, König J, Hutchinson AN, Thunberg P, Persson J (2022). Multi-strain probiotic mixture affects brain morphology and resting state brain function in healthy subjects: An RCT. Cells..

[44] Messaoudi M, Lalonde R, Violle N, Javelot H, Desor D, Nejdi A (2011). Assessment of psychotropic-like properties of a probiotic formulation (Lactobacillus helveticus R0052 and Bifidobacterium longum R0175) in rats and human subjects. Br. J. Nutr..

[45] Norlin AK, Walter S, Theodorsson E, Tegelstrom V, Grodzinsky E, Jones MP (2017). Cortisol levels in hair are altered in irritable bowel syndrome-A case control study in primary care. J. Psychosom. Res..

[46] Herr N, Bode C, Duerschmied D (2017). The effects of serotonin in immune cells. Front. Cardiovasc. Med..

[47] Kwon YH, Wang H, Denou E, Ghia JE, Rossi L, Fontes ME, Bernier SP, Shajib MS, Banskota S, Collins SM, Surette MG (2019). Modulation of gut microbiota composition by serotonin signaling influences intestinal immune response and susceptibility to colitis. Cell. Mol. Gastroenterol. Hepatol..

[48] Vahora IS, Tsouklidis N, Kumar R, Soni R, Khan S (2020). How serotonin level fluctuation affects the effectiveness of treatment in irritable bowel syndrome. Cureus..

[49] Ayob N, Muhammad Nawawi KN, Mohamad Nor MH, Raja Ali RA, Ahmad HF, Oon SF (2023). The effects of probiotics on small intestinal microbiota composition, inflammatory cytokines and intestinal permeability in patients with non-alcoholic fatty liver disease. Biomedicines..

[50] Ranadheera CS, Vidanarachchi JK, Rocha RS, Cruz AG, Ajlouni S (2017). Probiotic delivery through fermentation: Dairy vs non-dairy beverages. Fermentation..

[51] Yee A, Hodori NA, Tung YZ, Ooi PL, Latif SA, Isa HM (2021). Depression level and coping responses toward the movement control order and its impact on quality of life in the Malaysian community during the COVID-19 pandemic: A web-based cross-sectional study. Ann. Gen. Psychiatry..

[52] Wan Mohd Yunus WM, Badri SK, Panatik SA, Mukhtar F (2021). The unprecedented movement control order (lockdown) and factors associated with the negative emotional symptoms, happiness, and work-life balance of Malaysian University students during the coronavirus disease (COVID-19) pandemic. Front. Psychiatr..

[53] Bahrudin MF, Abdul Rani R, Tamil AM, Mokhtar NM, Raja Ali RA (2020). Effectiveness of sterilized symbiotic drink containing Lactobacillus helveticus comparable to probiotic alone in patients with constipation-predominant irritable bowel syndrome. Digest. Dis. Sci..

[54] Shafiee NH, Razalli NH, Mokhtar NM, Tan E, Ali RA (2022). An evaluation of dietary adequacy among patients with constipation-predominant irritable bowel syndrome in Malaysia. Intest. Res..

[55] Wong Z, Mok CZ, Majid HA, Mahadeva S (2018). Early experience with a low FODMAP diet in Asian patients with irritable bowel syndrome. JGH Open..

