# Supplementary material for: A randomized, double-blinded, placebo-controlled clinical trial on Lactobacillus-containing cultured milk drink as adjuvant therapy for depression in irritable bowel syndrome
Source: Sci Rep. 2024 Apr 25;14:9478. doi: 10.1038/s41598-024-60029-2 (PMC11043363; doi:10.1038/s41598-024-60029-2)
Supplement: Supplementary file 1 — Supplementary Table 1. [file 41598_2024_60029_MOESM1_ESM.docx]

**Supplementary Table 1S.** Comparison of baseline measures between IBS participants with subthreshold depression and those with normal mood (n=110).

| Variables | Normal mood, NM  (CESD-R<16) (n=57) | Subthreshold depression, SD  (CESD-R≥16)  (n=53) | p-value |
| --- | --- | --- | --- |
| Depression risk (Mean ± s.d.) | | | |
| CESD-R score | 5.0 **±** 8.0 | 21.0 **±** 9.5 | <0.001** |
| Quality of life (Median, IQR) | | | |
| Total IBS-QOL score | 522.3(113.2) | 430.1(197.5) | 0.005* |
| Domain 1: Dysphoria | 90.6(12.5) | 78.1(39.1) | 0.007* |
| Domain 2: Interference with activity | 82.1(23.2) | 71.1 (39.3) | 0.043* |
| Domain 3: Body image | 87.5(21.9) | 68.8 (43.8) | 0.006* |
| Domain 4: Health worry | 83.3(25.0) | 58.3 (41.7) | 0.012* |
| Domain 5: Food avoidance | 75.0(33.3) | 66.7 (41.7) | 0.027* |
| Domain 6: Social reaction | 81.3(25.0) | 75.0 (37.5) | 0.031* |
| Domain 7: Relationship | 91.7(25.0) | 83.3 (33.3) | 0.003* |
| Domain 8: Sexual | 100.0(0.0) | 100.0 (18.8) | 0.105 |
| Hormone serum level (ng/ml) (Mean ± s.d.) | | | |
| Cortisol | 241.1 **±** 200.7 | 260.0 **±** 229.9 | 0.740 |
| Serotonin | 190.7 ± 314.7 | 153.3 ± 128.9 | 0.561 |
| IBS symptoms severity (Mean ± s.d.) | | | |
| Total IBS-SSS Score | 204.3 **±** 74.4 | 236.3 **±** 81.5 | 0.033* |
| Subscale 1: Abdominal pain severity | 42.3 **±** 21.5 | 49.3 **±** 22.0 | 0.096 |
| Subscale 2: Number of days with abdominal pain | 38.8 **±** 27.4 | 44.4 **±** 29.7 | 0.301 |
| Subscale 3: Abdominal distension | 38.3 **±** 19.9 | 38.8 **±** 20.6 | 0.910 |
| Subscale 4: Bowel habit dissatisfaction | 44.4 **±** 19.7 | 54.4 **±** 21.8 | 0.013* |
| Subscale 5: Life disruption | 40.7 ± 20.3 | 49.4 ± 22.4 | 0.034* |
| IBS severity category, *n* (%) |  |  |  |
| Remission (<75) | 2(3.5) | 0 | 0.039* |
| Mild (75-174) | 16(28.1) | 9(17.0) |  |
| Moderate (175-299) | 32(56.1) | 33(62.3) |  |
| Severe (≥300) | 7(12.3) | 11(20.8) |  |

Data are expressed as means ± standard deviations, medians (interquartile range) or percentages (%) based on data distribution. p-value obtained from independent t-test and Mann-Whitney U test for non-parametric data. (*p<0.05, **p<0.001). IQR, interquartile range; s.d, standard deviation.
