# Supplementary material for: A randomized, double-blinded, placebo-controlled clinical trial on Lactobacillus-containing cultured milk drink as adjuvant therapy for depression in irritable bowel syndrome
Source: Sci Rep. 2024 Apr 25;14:9478. doi: 10.1038/s41598-024-60029-2 (PMC11043363; doi:10.1038/s41598-024-60029-2)
Supplement: Supplementary file 2 — Supplementary Table 2. [file 41598_2024_60029_MOESM2_ESM.docx]

**Supplementary Table 2S.** CESD-R scores (log mean) at pre- and post-intervention.

| **Parameter** | **Group** |  | **Sample size** | **Mean** | **SD** | **MD**  **(95% CI)** | **p-value** | **Effect size** |
| --- | --- | --- | --- | --- | --- | --- | --- | --- |
| CESD-R score (log mean) | IBS-NM with placebo | Baseline | 29 | 0.74 | 0.31 | -0.02  (-0.28, 0.11) | 0.806 | 0.16 |
|  |  | End of trial | 29 | 0.76 | 0.41 |  |  |  |
|  | IBS-NM with probiotic | Baseline | 28 | 0.69 | 0.34 | 0.09  (-0.12, 0.21) | 0.264 | 0.11 |
|  |  | End of trial | 28 | 0.60 | 0.47 |  |  |  |
|  | IBS-SD with placebo | Baseline | 27 | 1.35 | 0.12 | 0.46  (0.29, 0.63) | 0.000* | 1.10 |
|  |  | End of trial | 27 | 0.89 | 0.42 |  |  |  |
|  | IBS-SD with probiotic | Baseline | 26 | 1.37 | 0.13 | 0.31  (0.19, 0.43) | 0.000* | 1.05 |
|  |  | End of trial | 26 | 1.07 | 0.34 |  |  |  |

Data expressed in mean ± standard deviation. Data was analysed with paired t-test where * represents p-value <0.05. SD, standard deviation; MD, mean difference; CESD-R, Center for Epidemiologic Studies Depression Revised; IBS-NM, irritable bowel syndrome with normal mood; IBS-SD, irritable bowel syndrome with subthreshold depression.
