# Supplementary material for: A randomized, double-blinded, placebo-controlled clinical trial on Lactobacillus-containing cultured milk drink as adjuvant therapy for depression in irritable bowel syndrome
Source: Sci Rep. 2024 Apr 25;14:9478. doi: 10.1038/s41598-024-60029-2 (PMC11043363; doi:10.1038/s41598-024-60029-2)
Supplement: Supplementary file 3 — Supplementary Table 3. [file 41598_2024_60029_MOESM3_ESM.docx]

**Supplementary Table 3S.** Distribution of normal mood and subthreshold depression across all groups at post-intervention.

| Changes in the distribution | Normal mood,  n (%) | Subthreshold depression,  n (%) |
| --- | --- | --- |
| IBS-NM with placebo | 27 (93.1) | 2 (6.9) |
| IBS-NM with probiotic | 25 (89.3) | 3 (10.7) |
| IBS-SD with placebo | 20 (74.1) | 7 (25.9) |
| IBS-SD with probiotic | 17 (65.4) | 9 (34.6) |
| Overall | 89 (80.9) | 21 (19.1) |

Data expressed in frequency and percetage (%). Data was analysed with descriptive statistical analysis. n, frequency; IBS-NM, irritable bowel syndrome with normal mood; IBS-SD, irritable bowel syndrome with subthreshold depression.
