# Supplementary material for: A randomized, double-blinded, placebo-controlled clinical trial on Lactobacillus-containing cultured milk drink as adjuvant therapy for depression in irritable bowel syndrome
Source: Sci Rep. 2024 Apr 25;14:9478. doi: 10.1038/s41598-024-60029-2 (PMC11043363; doi:10.1038/s41598-024-60029-2)
Supplement: Supplementary file 4 — Supplementary Table 4. [file 41598_2024_60029_MOESM4_ESM.docx]

**Supplementary Table 4S.** Changes in IBS-QOL scores among IBS participants.

| **Parameter** | **Group** |  | **Sample size** | **Median** | **IQR** | | **p_1_** | **p_2_** |
| --- | --- | --- | --- | --- | --- | --- | --- | --- |
|  |  |  |  |  | **Q1** | **Q3** |  |  |
| ∑IBS-QOL | IBS-NM with placebo | Baseline | 29 | 80.88 | 70.59 | 91.54 | 0.031* | 0.044* |
|  |  | End of trial | 29 | 88.97 | 78.31 | 96.32 |  |  |
|  | IBS-NM with probiotic | Baseline | 28 | 86.77 | 77.02 | 94.85 | 0.355 |  |
|  |  | End of trial | 28 | 91.54 | 72.98 | 96.51 |  |  |
|  | IBS-SD placebo | Baseline | 27 | 66.91 | 50.00 | 85.29 | 0.002* |  |
|  |  | End of trial | 27 | 84.56 | 66.18 | 97.79 |  |  |
|  | IBS-SD with probiotic | Baseline | 26 | 77.57 | 64.34 | 89.71 | 0.019* |  |
|  |  | End of trial | 26 | 83.09 | 75.00 | 32.83 |  |  |
| Dysphoria | IBS-NM with placebo | Baseline | 29 | 84.38 | 78.13 | 93.75 | 0.221 | 0.108 |
|  |  | End of trial | 29 | 90.63 | 73.44 | 98.44 |  |  |
|  | IBS-NM with probiotic | Baseline | 28 | 90.63 | 81.25 | 96.88 | 0.760 |  |
|  |  | End of trial | 28 | 93.75 | 84.38 | 96.88 |  |  |
|  | IBS-SD placebo | Baseline | 27 | 65.63 | 43.75 | 90.63 | 0.010* |  |
|  |  | End of trial | 27 | 84.38 | 65.63 | 100.00 |  |  |
|  | IBS-SD with probiotic | Baseline | 26 | 84.38 | 68.75 | 93.75 | 0.039* |  |
|  |  | End of trial | 26 | 90.63 | 75.00 | 96.88 |  |  |
| Interference to activities | IBS-NM with placebo | Baseline | 29 | 82.14 | 58.93 | 92.86 | 0.208 | 0.274 |
|  |  | End of trial | 29 | 89.29 | 75.00 | 94.64 |  |  |
|  | IBS-NM with probiotic | Baseline | 28 | 83.93 | 73.21 | 92.86 | 0.282 |  |
|  |  | End of trial | 28 | 89.29 | 71.43 | 96.43 |  |  |
|  | IBS-SD placebo | Baseline | 27 | 67.86 | 42.86 | 89.29 | 0.014* |  |
|  |  | End of trial | 27 | 82.14 | 57.14 | 96.43 |  |  |
|  | IBS-SD with probiotic | Baseline | 26 | 75.00 | 57.14 | 90.18 | 0.091 |  |
|  |  | End of trial | 26 | 78.57 | 67.86 | 92.86 |  |  |
| Body image | IBS-NM with placebo | Baseline | 29 | 81.25 | 68.75 | 96.88 | 0.125 | 0.511 |
|  |  | End of trial | 29 | 87.50 | 81.25 | 93.75 |  |  |
|  | IBS-NM with probiotic | Baseline | 28 | 87.50 | 76.56 | 93.75 | 0.420 |  |
|  |  | End of trial | 28 | 93.75 | 70.31 | 93.75 |  |  |
|  | IBS-SD placebo | Baseline | 27 | 62.50 | 43.75 | 87.50 | 0.019* |  |
|  |  | End of trial | 27 | 81.25 | 50.00 | 93.75 |  |  |
|  | IBS-SD with probiotic | Baseline | 26 | 75.00 | 62.50 | 93.75 | 0.084 |  |
|  |  | End of trial | 26 | 81.25 | 75.00 | 100.00 |  |  |
| Health worry | IBS-NM with placebo | Baseline | 29 | 75.00 | 66.67 | 91.67 | 0.175 | 0.007* |
|  |  | End of trial | 29 | 83.33 | 66.67 | 91.67 |  |  |
|  | IBS-NM with probiotic | Baseline | 28 | 83.33 | 52.08 | 91.67 | 0.153 |  |
|  |  | End of trial | 28 | 83.33 | 66.67 | 91.67 |  |  |
|  | IBS-SD placebo | Baseline | 27 | 50.00 | 33.33 | 75.00 | 0.000** |  |
|  |  | End of trial | 27 | 83.33 | 58.33 | 100.00 |  |  |
|  | IBS-SD with probiotic | Baseline | 26 | 70.83 | 47.92 | 91.67 | 0.018* |  |
|  |  | End of trial | 26 | 79.17 | 66.67 | 91.67 |  |  |
| Food avoidance | IBS-NM with placebo | Baseline | 29 | 58.33 | 58.33 | 83.33 | 0.010* | 0.151 |
|  |  | End of trial | 29 | 83.33 | 66.67 | 95.83 |  |  |
|  | IBS-NM with probiotic | Baseline | 28 | 83.33 | 66.67 | 97.92 | 0.119 |  |
|  |  | End of trial | 28 | 87.50 | 75.00 | 100.00 |  |  |
|  | IBS-SD placebo | Baseline | 27 | 58.33 | 33.33 | 83.33 | 0.003* |  |
|  |  | End of trial | 27 | 83.33 | 50.00 | 100.00 |  |  |
|  | IBS-SD with probiotic | Baseline | 26 | 66.67 | 50.00 | 83.33 | 0.040* |  |
|  |  | End of trial | 26 | 75.00 | 58.33 | 91.67 |  |  |
| Social reaction | IBS-NM with placebo | Baseline | 29 | 81.25 | 65.63 | 96.88 | 0.093 | 0.066 |
|  |  | End of trial | 29 | 87.50 | 78.13 | 100.00 |  |  |
|  | IBS-NM with probiotic | Baseline | 28 | 87.50 | 76.56 | 100.00 | 0.348 |  |
|  |  | End of trial | 28 | 93.75 | 81.25 | 100.00 |  |  |
|  | IBS-SD placebo | Baseline | 27 | 62.50 | 50.00 | 93.75 | 0.001* |  |
|  |  | End of trial | 27 | 93.75 | 68.75 | 100.00 |  |  |
|  | IBS-SD with probiotic | Baseline | 26 | 81.25 | 60.94 | 93.75 | 0.142 |  |
|  |  | End of trial | 26 | 81.25 | 75.00 | 93.75 |  |  |
| Relationship | IBS-NM with placebo | Baseline | 29 | 91.67 | 75.00 | 100.00 | 0.328 | 0.560 |
|  |  | End of trial | 29 | 91.67 | 87.50 | 100.00 |  |  |
|  | IBS-NM with probiotic | Baseline | 28 | 91.67 | 83.33 | 100.00 | 0.822 |  |
|  |  | End of trial | 28 | 91.67 | 75.00 | 100.00 |  |  |
|  | IBS-SD placebo | Baseline | 27 | 83.33 | 58.33 | 100.00 | 0.008* |  |
|  |  | End of trial | 27 | 91.67 | 75.00 | 100.00 |  |  |
|  | IBS-SD with probiotic | Baseline | 26 | 83.33 | 64.58 | 91.67 | 0.057 |  |
|  |  | End of trial | 26 | 91.67 | 75.00 | 100.00 |  |  |
| Sexual | IBS-NM with placebo | Baseline | 29 | 100.00 | 87.50 | 100.00 | 0.378 | 0.045* |
|  |  | End of trial | 29 | 100.00 | 87.50 | 100.00 |  |  |
|  | IBS-NM with probiotic | Baseline | 28 | 100.00 | 100.00 | 100.00 | 0.792 |  |
|  |  | End of trial | 28 | 100.00 | 90.63 | 100.00 |  |  |
|  | IBS-SD placebo | Baseline | 27 | 100.00 | 75.00 | 100.00 | 0.081 |  |
|  |  | End of trial | 27 | 100.00 | 87.50 | 100.00 |  |  |
|  | IBS-SD with probiotic | Baseline | 26 | 100.00 | 87.50 | 100.00 | 0.291 |  |
|  |  | End of trial | 26 | 100.00 | 87.50 | 100.00 |  |  |

Data expressed in median and interquartile range (IQR). Data was analysed with Wilcoxon Signed-Rank test for comparison within group and Kruskal-Wallis for evaluation of between group where * represents p-value <0.05. p_1_ is the p-value for within group analysis and p_2_ is the p-value for between group analysis. IQR, interquartile range; Q1, first quartile; Q3, third quartile; ∑, total sum; IBS-QOL, irritable bowel syndrome quality of life; IBS-NM, irritable bowel syndrome with normal mood; IBS-SD, irritable bowel syndrome with subthreshold depression.
