# Supplementary material for: A randomized, double-blinded, placebo-controlled clinical trial on Lactobacillus-containing cultured milk drink as adjuvant therapy for depression in irritable bowel syndrome
Source: Sci Rep. 2024 Apr 25;14:9478. doi: 10.1038/s41598-024-60029-2 (PMC11043363; doi:10.1038/s41598-024-60029-2)
Supplement: Supplementary file 5 — Supplementary Table 5. [file 41598_2024_60029_MOESM5_ESM.docx]

**Supplementary Table 5S.** IBS-SSS scores at pre- and post-intervention

| **Parameter** | **Group** | | **n** | **Mean** | **SD** | **MD**  **(95% CI)** | **p-value** | **Effect size (d)** |
| --- | --- | --- | --- | --- | --- | --- | --- | --- |
| ∑IBS-SSS | IBS-NM with placebo | Baseline | 29 | 205.52 | 68.54 | 61.55  (20.74, 102.37) | 0.004* | 0.57 |
|  |  | End of trial | 29 | 143.97 | 84.40 |  |  |  |
|  | IBS-NM with probiotic | Baseline | 28 | 203.04 | 81.26 | 55.18  (3.64, 106.71) | 0.037* | 0.42 |
|  |  | End of trial | 28 | 147.86 | 94.23 |  |  |  |
|  | IBS-SD with placebo | Baseline | 27 | 235.93 | 70.28 | 64.44  (24.68, 104.21) | 0.003* | 0.64 |
|  |  | End of trial | 27 | 171.48 | 79.01 |  |  |  |
|  | IBS-SD with probiotic | Baseline | 26 | 236.73 | 93.11 | 99.62  (49.11, 150.13) | 0.000# | 0.80 |
|  |  | End of trial | 26 | 137.12 | 75.09 |  |  |  |
| Abdominal pain severity | IBS-NM with placebo | Baseline | 29 | 43.79 | 19.53 | 12.76  (3.67, 21.85) | 0.008* | 0.53 |
|  |  | End of trial | 29 | 31.03 | 20.59 |  |  |  |
|  | IBS-NM with probiotic | Baseline | 28 | 40.71 | 23.56 | 6.79  (-0.61, 14.18) | 0.000** | 0.36 |
|  |  | End of trial | 28 | 33.93 | 23.62 |  |  |  |
|  | IBS-SD with placebo | Baseline | 27 | 49.26 | 20.37 | 22.41  (14.62, 30.19) | 0.000** | 1.14 |
|  |  | End of trial | 27 | 26.85 | 20.72 |  |  |  |
|  | IBS-SD with probiotic | Baseline | 26 | 49.23 | 23.99 | 9.23  (2.01, 16.45) | 0.014* | 0.52 |
|  |  | End of trial | 26 | 40.00 | 19.18 |  |  |  |
| Number of days with abdominal pain | IBS-NM with placebo | Baseline | 29 | 36.38 | 24.75 | 16.72  (6.08, 27.37) | 0.003* | 0.60 |
|  |  | End of trial | 29 | 19.66 | 26.69 |  |  |  |
|  | IBS-NM with probiotic | Baseline | 28 | 41.25 | 30.11 | 18.93  (9.21, 28.65) | 0.000** | 0.76 |
|  |  | End of trial | 28 | 22.32 | 25.26 |  |  |  |
|  | IBS-SD with placebo | Baseline | 27 | 43.33 | 29.94 | 24.63  (17.46, 31.80) | 0.000** | 1.36 |
|  |  | End of trial | 27 | 18.70 | 22.43 |  |  |  |
|  | IBS-SD with probiotic | Baseline | 26 | 45.58 | 30.08 | 16.35  (6.07, 26.62) | 0.003* | 0.64 |
|  |  | End of trial | 26 | 29.23 | 28.13 |  |  |  |
| Abdominal distension | IBS-NM with placebo | Baseline | 29 | 40.86 | 20.53 | 10.17  (0.99, 19.36) | 0.031* | 0.42 |
|  |  | End of trial | 29 | 30.69 | 21.54 |  |  |  |
|  | IBS-NM with probiotic | Baseline | 28 | 35.71 | 19.33 | 8.93  (3.94, 13.92) | 0.001* | 0.69 |
|  |  | End of trial | 28 | 26.79 | 14.42 |  |  |  |
|  | IBS-SD with placebo | Baseline | 27 | 35.00 | 19.76 | 11.85  (1.84, 21.87) | 0.022* | 0.47 |
|  |  | End of trial | 27 | 23.15 | 20.34 |  |  |  |
|  | IBS-SD with probiotic | Baseline | 26 | 42.68 | 21.08 | 5.77  (-1.82, 13.36) | 0.130 | 0.31 |
|  |  | End of trial | 26 | 36.92 | 21.68 |  |  |  |
| Bowel habit dissatisfaction | IBS-NM with placebo | Baseline | 29 | 43.10 | 19.43 | 20.00  (11.49, 28.51) | 0.000** | 0.89 |
|  |  | End of trial | 29 | 23.10 | 20.59 |  |  |  |
|  | IBS-NM with probiotic | Baseline | 28 | 45.71 | 20.26 | 6.79  (-3.00, 16.58) | 0.166 | 0.27 |
|  |  | End of trial | 28 | 38.93 | 27.26 |  |  |  |
|  | IBS-SD with placebo | Baseline | 27 | 57.22 | 20.68 | 30.00  (19.38, 40.62) | 0.000** | 1.12 |
|  |  | End of trial | 27 | 27.22 | 24.59 |  |  |  |
|  | IBS-SD with probiotic | Baseline | 26 | 51.54 | 22.92 | 13.08  (4.47, 21.68) | 0.004* | 0.61 |
|  |  | End of trial | 26 | 38.46 | 25.41 |  |  |  |
| Life disruption | IBS-NM with placebo | Baseline | 29 | 41.38 | 18.27 | 16.03  (9.48, 22.59) | 0.000** | 0.93 |
|  |  | End of trial | 29 | 25.35 | 18.27 |  |  |  |
|  | IBS-NM with probiotic | Baseline | 28 | 40.00 | 22.44 | 7.86  (-0.20, 15.92) | 0.056 | 0.38 |
|  |  | End of trial | 28 | 32.14 | 26.44 |  |  |  |
|  | IBS-SD with placebo | Baseline | 27 | 51.11 | 20.25 | 15.56  (7.03, 24.08) | 0.001* | 0.72 |
|  |  | End of trial | 27 | 35.56 | 22.93 |  |  |  |
|  | IBS-SD with probiotic | Baseline | 26 | 47.69 | 24.71 | 6.54  (-1.70, 14.77) | 0.114 | 0.32 |
|  |  | End of trial | 26 | 41.15 | 28.19 |  |  |  |

Data expressed in mean ± standard deviation. Data was analysed with paired t-test where * represents p-value <0.05 and ** represents p-value <0.001. n, sample size; SD, standard deviation; MD, mean difference; CI, confidence interval; ∑, total sum; IBS-NM, irritable bowel syndrome with normal mood; IBS-SD, irritable bowel syndrome with subthreshold depression; IBS-SSS, irritable bowel syndrome severity scoring system.
