# Supplementary material for: A randomized, double-blinded, placebo-controlled clinical trial on Lactobacillus-containing cultured milk drink as adjuvant therapy for depression in irritable bowel syndrome
Source: Sci Rep. 2024 Apr 25;14:9478. doi: 10.1038/s41598-024-60029-2 (PMC11043363; doi:10.1038/s41598-024-60029-2)
Supplement: Supplementary file 6 — Supplementary Table 6. [file 41598_2024_60029_MOESM6_ESM.docx]

**Supplementary Table 6S.** Distribution of IBS severity during pre- and post-intervention.

| IBS symptoms severity |  | IBS-NM with placebo, n (%) | IBS-NM with probiotic, n (%) | IBS-SD with placebo, n (%) | IBS-SD with probiotic, n (%) | p-value |
| --- | --- | --- | --- | --- | --- | --- |
| Remission  (<75) | Baseline | 0 (0) | 2 (7.1) | 0 (0) | 0 (0) | 0.000# |
|  | End of trial | 8 (27.6) | 3 (10.7) | 5 (18.5) | 4 (15.4) |  |
|  | Difference | 27.6% | 3.6% | 18.5% | 15.4% |  |
| Mild  (75-174) | Baseline | 8 (27.6) | 8 (28.6) | 5 (18.5) | 4 (15.4) | 0.005* |
|  | End of trial | 14 (48.3) | 15 (53.6) | 16 (59.3) | 10 (38.5) |  |
|  | Difference | 20.7% | 25% | 40.8% | 23.1% |  |
| Moderate  (175-299) | Baseline | 18 (62.1) | 14 (50.0) | 16 (59.3) | 17 (65.4) | 0.000# |
|  | End of trial | 7 (24.1) | 9 (32.1) | 5 (18.5) | 8 (30.8) |  |
|  | Difference | -38% | -17.9% | -40.8% | -34.6% |  |
| Severe  (≥300) | Baseline | 3 (10.3) | 4 (14.3) | 6 (22.2) | 5 (19.2) | 0.002* |
|  | End of trial | 0 (0) | 1 (3.6) | 1 (3.7) | 4 (15.4) |  |
|  | Difference | 10.3% | -10.7% | -18.5% | -3.8% |  |

Data expressed in mean ± standard deviation. Data was analysed with paired t-test where * represents p-value <0.05 and # represents p-value <0.001. n, sample size; SD, standard deviation; MD, mean difference; CI, confidence interval; ∑, total sum; IBS-NM, irritable bowel syndrome with normal mood; IBS-SD, irritable bowel syndrome with subthreshold depression; IBS-SSS, irritable bowel syndrome severity scoring system.
