# Supplementary material for: A randomized, double-blinded, placebo-controlled clinical trial on Lactobacillus-containing cultured milk drink as adjuvant therapy for depression in irritable bowel syndrome
Source: Sci Rep. 2024 Apr 25;14:9478. doi: 10.1038/s41598-024-60029-2 (PMC11043363; doi:10.1038/s41598-024-60029-2)
Supplement: Supplementary file 7 — Supplementary Table 7. [file 41598_2024_60029_MOESM7_ESM.docx]

**Supplementary Table 7S.** Hormonal changes with cultured milk drink intervention (n=58).

| **Hormones** | **Group** | | **Sample size** | **Mean** | **SD** | **MD**  **(95% CI)** | **p-value** | **Effect size** |
| --- | --- | --- | --- | --- | --- | --- | --- | --- |
| Cortisol (log mean) | IBS-NM with placebo | Baseline | 19 | 2.13 | 0.34 | -0.22  (-0.37, 0.06) | .008* | 0.68 |
|  |  | End of trial | 19 | 2.35 | 0.35 |  |  |  |
|  | IBS-NM with probiotic | Baseline | 11 | 2.40 | 0.44 | -0.07  (-0.23, 0.09) | .369 | 0.28 |
|  |  | End of trial | 11 | 2.47 | 0.31 |  |  |  |
|  | IBS-SD with placebo | Baseline | 15 | 2.23 | 0.20 | -0.10  (-0.23, 0.04) | .139 | 0.41 |
|  |  | End of trial | 15 | 2.33 | 0.29 |  |  |  |
|  | IBS-SD with probiotic | Baseline | 13 | 2.28 | 0.54 | -0.14  (-0.31, 0.02) | .086 | 0.52 |
|  |  | End of trial | 13 | 2.43 | 0.36 |  |  |  |
| 5HT (log mean) | IBS-NM with placebo | Baseline | 19 | 1.92 | 0.31 | -0.09  (-0.21, 0.02) | .108 | 0.39 |
|  |  | End of trial | 19 | 2.00 | 0.37 |  |  |  |
|  | IBS-NM with probiotic | Baseline | 11 | 2.23 | 0.46 | -0.01  (-0.30, 0.28) | .943 | 0.02 |
|  |  | End of trial | 11 | 2.24 | 0.32 |  |  |  |
|  | IBS-SD with placebo | Baseline | 15 | 1.99 | 0.37 | -0.09  (-0.22, 0.04) | .172 | 0.37 |
|  |  | End of trial | 15 | 2.08 | 0.38 |  |  |  |
|  | IBS-SD with probiotic | Baseline | 13 | 2.13 | 0.31 | -0.17  (-0.26, -0.08) | .002* | 1.13 |
|  |  | End of trial | 13 | 2.29 | 0.312 |  |  |  |

Data expressed in mean ± standard deviation. Data was analysed with paired t-test where * represents p-value <0.05. SD, standard deviation; IBS-NM, irritable bowel syndrome with normal mood; IBS-SD, irritable bowel syndrome with subthreshold depression; 5-HT serotonin.
