# Supplementary material for: A randomized, double-blinded, placebo-controlled clinical trial on Lactobacillus-containing cultured milk drink as adjuvant therapy for depression in irritable bowel syndrome
Source: Sci Rep. 2024 Apr 25;14:9478. doi: 10.1038/s41598-024-60029-2 (PMC11043363; doi:10.1038/s41598-024-60029-2)
Supplement: Supplementary file 8 — Supplementary Table 8. [file 41598_2024_60029_MOESM8_ESM.docx]

**Supplementary Table 8S.** Parameter comparison between groups after intervention.

| **Parameter** | **ANOVA** | | | **ANCOVA** | | |
| --- | --- | --- | --- | --- | --- | --- |
|  | **MD**  **(95% CI)** | **p_1_** | **Effect size** | **MD**  **(95% CI)** | **p_2_** | **Effect size** |
| **Depression symptoms scores** | | | |  | | |
| CESD-R (log mean) | 0.21 (0.13,0.28) | 0.000# | 0.434 | 0.20 (0.12, 0.28) | 0.000# | 0.304 |
| **Hormone serum level** | | | |  | | |
| Cortisol (log mean) | -0.13 (-0.21, -0.06) | 0.438 | 0.049 | -0.13 (-0.21, -0.06) | 0.456 | 0.048 |
| 5-HT (log mean) | -0.09 (-0.16, -0.02) | 0.075 | 0.119 | -0.09 (-0.16, -0.02) | 0.074 | 0.122 |
| **IBS Sympotoms Severity** | | | |  | | |
| ∑IBS-SSS | 69.58 (56.71, 82.44) | 0.170 | 0.046 | 69.64 (56.28, 82.99) | 0.012* | 0.098 |
| Abdominal pain severity | 12.80 (8.95, 16.64) | 0.435 | 0.025 | 12.77 (8.82, 16.73) | 0.037* | 0.078 |
| Number of days with abdominal pain | 19.16 (14.52, 23.79) | 0.554 | 0.019 | 19.14 (14.53, 23.74) | 0.593 | 0.018 |
| Abdominal distension | 9.18 (5.23, 13.13) | 0.102 | 0.057 | 9.23 (5.31, 13.14) | 0.744 | 0.012 |
| Bowel habit dissatisfaction | 17.47 (12.92, 22.02) | 0.112 | 0.055 | 17.46 (12.69, 22.22) | 0.005* | 0.116 |
| Life disruption | 11.50 (7.71, 15.28) | 0.130 | 0.052 | 11.59 (7.77, 15.41) | 0.169 | 0.047 |

Data expressed in mean ± standard deviation. Data was analysed with GLM ANOVA repeated measures and ANCOVA with adjustment to baseline covariate. The p1 is the p-value between groups and p2 is the p-value with covariate adjustment. * represents p-value <0.05. GLM, general linear model; CI, confidence interval; CESDR, Center of Epidemiologic Studies Depression Revised; SD, standard deviation; IBS-NM, irritable bowel syndrome with normal mood; IBS-SD, irritable bowel syndrome with subthreshold depression; 5-HT serotonin; ∑, total; IBS-SSS, *irritable bowel syndrome severity scoring system*.
