# Supplementary material for: A randomized, double-blinded, placebo-controlled clinical trial on Lactobacillus-containing cultured milk drink as adjuvant therapy for depression in irritable bowel syndrome
Source: Sci Rep. 2024 Apr 25;14:9478. doi: 10.1038/s41598-024-60029-2 (PMC11043363; doi:10.1038/s41598-024-60029-2)
Supplement: Supplementary file 9 — Supplementary Table 9. [file 41598_2024_60029_MOESM9_ESM.docx]

**Supplementary Table 9S.** CESD-R changes comparison between groups.

| **Parameter** | **Group** | | **Mean difference** | **95% CI** | | **p-value** |
| --- | --- | --- | --- | --- | --- | --- |
|  |  |  |  | **Lower limit** | **Upper limit** |  |
| CESD-R (log mean) | IBS-NM with placebo | IBS-SD with placebo | -0.366 | -0.566 | -0.165 | <.001# |
|  |  | IBS-SD with probiotic | -0.467 | 0.67 | -0.265 | <.001# |
|  | IBS-NM with probiotic | IBS-SD with placebo | -0.473 | -0.675 | -0.270 | <.001# |
|  |  | IBS-SD with probiotic | -0.574 | -0.779 | -0.370 | <.001# |

Data expressed in mean ± standard deviation. Data was analysed with GLM ANOVA repeated measures and Boferroni post hoc analysis. #represents p-value <0.001. GLM, general linear model; CI, confidence interval; CESDR, Center of Epidemiologic Studies Depression Revised; SD, standard deviation; IBS-NM, irritable bowel syndrome with normal mood; IBS-SD, irritable bowel syndrome with subthreshold depression.
