# Supplementary material for: A randomized, double-blinded, placebo-controlled clinical trial on Lactobacillus-containing cultured milk drink as adjuvant therapy for depression in irritable bowel syndrome
Source: Sci Rep. 2024 Apr 25;14:9478. doi: 10.1038/s41598-024-60029-2 (PMC11043363; doi:10.1038/s41598-024-60029-2)
Supplement: Supplementary file 10 — Supplementary Table 10. [file 41598_2024_60029_MOESM10_ESM.docx]

**Supplementary Table 10S.** IBS-QOL changes comparison between groups with Mann-Whitney U-test.

| **Groups** | | **IBS-QOL domain (score)** | | |
| --- | --- | --- | --- | --- |
|  |  | ∑**IBS-QOL** | **Health worry** | **Sexual** |
| IBS-NM placebo | IBS-SD placebo | Z = -1.959  U = 272  p = 0.049* | Z = -3.108  U = 204  p = 0.002* |  |
| IBS-NM probiotic | IBS-SD placebo | Z = -2.576  U = 225  p = 0.01* | Z = -2.887  U = 208.5  p = 0.004* | Z = -2.760  U = 217.5  p = 0.006* |

Data expressed in mean ± standard deviation. Data was analysed with Mann-Whitney U-test, where * represents p-value <0.05. IBS-NM, irritable bowel syndrome with normal mood; IBS-SD, irritable bowel syndrome with subthreshold depression; ∑, overall; IBS-QOL, irritable bowel syndrome quality of life.
