# Supplementary material for: A randomized, double-blinded, placebo-controlled clinical trial on Lactobacillus-containing cultured milk drink as adjuvant therapy for depression in irritable bowel syndrome
Source: Sci Rep. 2024 Apr 25;14:9478. doi: 10.1038/s41598-024-60029-2 (PMC11043363; doi:10.1038/s41598-024-60029-2)
Supplement: Supplementary file 11 — Supplementary Table 11. [file 41598_2024_60029_MOESM11_ESM.docx]

**Supplementary Table 11S.** IBS-SSS changes comparison between groups with covariate adjustment.

| **Groups** | | **IBS-SSS subscale** | **Mean difference** | **95% CI** | | **p-value** |
| --- | --- | --- | --- | --- | --- | --- |
|  |  |  |  | **Lower limit** | **Upper limit** |  |
| IBS-NM probiotic | IBS-SD placebo | ∑IBS-SSS | -53.428 | -103.15 | -3.697 | .0028* |
|  |  | Abdominal pain severity | -15.057 | -29.934 | -0.167 | .046* |
|  |  | Bowel habit dissatisfaction | -23.186 | -40.846 | -5.525 | .004* |
| IBS-SD placebo | IBS-SD probiotic | ∑IBS-SSS | -53.981 | -104.278 | -3.685 | .028* |

Data expressed in mean ± standard deviation. Data was analysed with ANCOVA with covariate adjustment (gender), where * represents p-value <0.05. IBS-NM, irritable bowel syndrome with normal mood; IBS-SD, irritable bowel syndrome with subthreshold depression; ∑, overall; IBS-SSS, irritable bowel syndrome severity scoring system.
