# Supplementary figures and images for: A randomized, double-blinded, placebo-controlled clinical trial on Lactobacillus-containing cultured milk drink as adjuvant therapy for depression in irritable bowel syndrome
Source: Sci Rep. 2024 Apr 25;14:9478. doi: 10.1038/s41598-024-60029-2 (PMC11043363; doi:10.1038/s41598-024-60029-2)

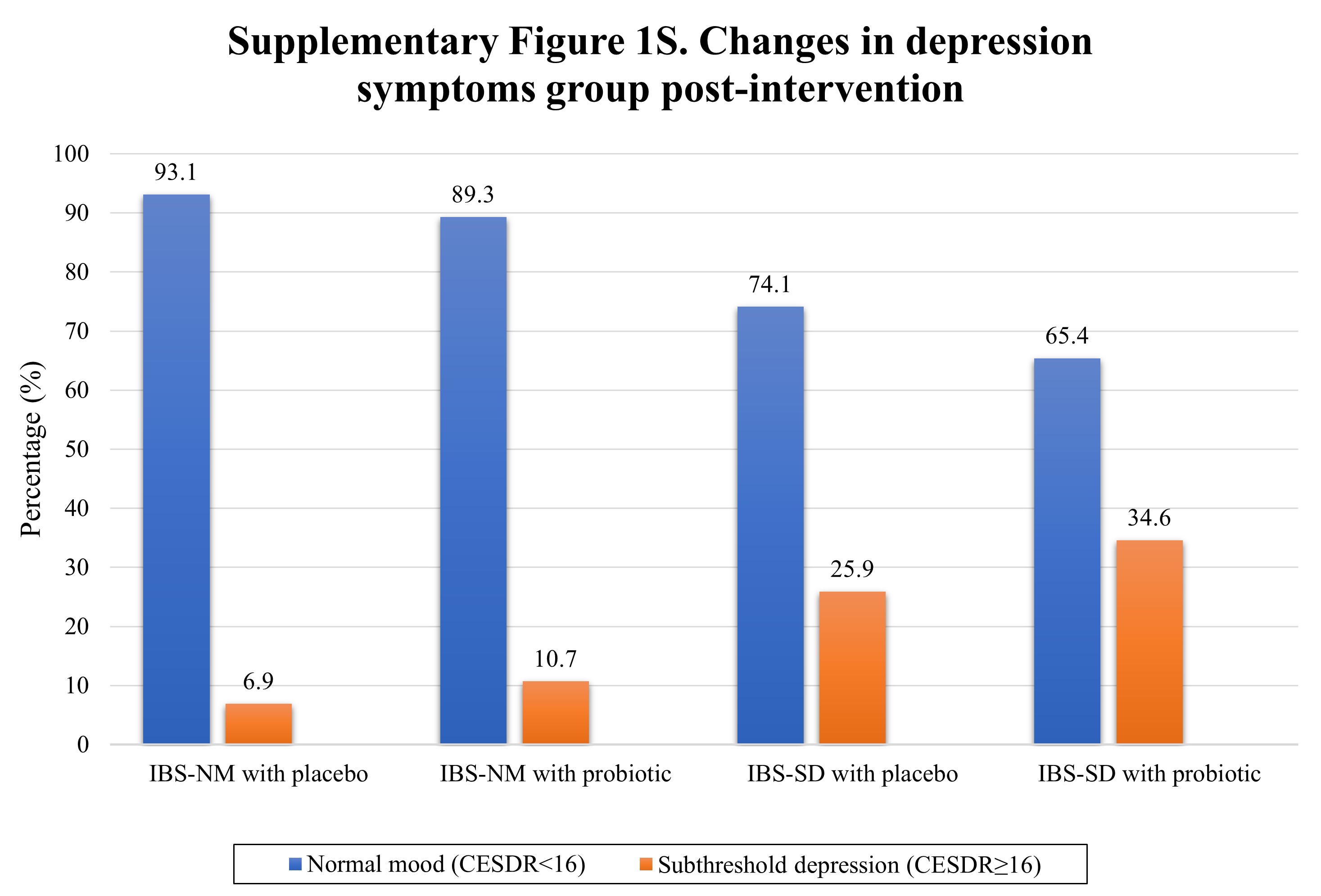

Supplement: Supplementary file 12 — Supplementary Figure 1. [file 41598_2024_60029_MOESM12_ESM.jpg]

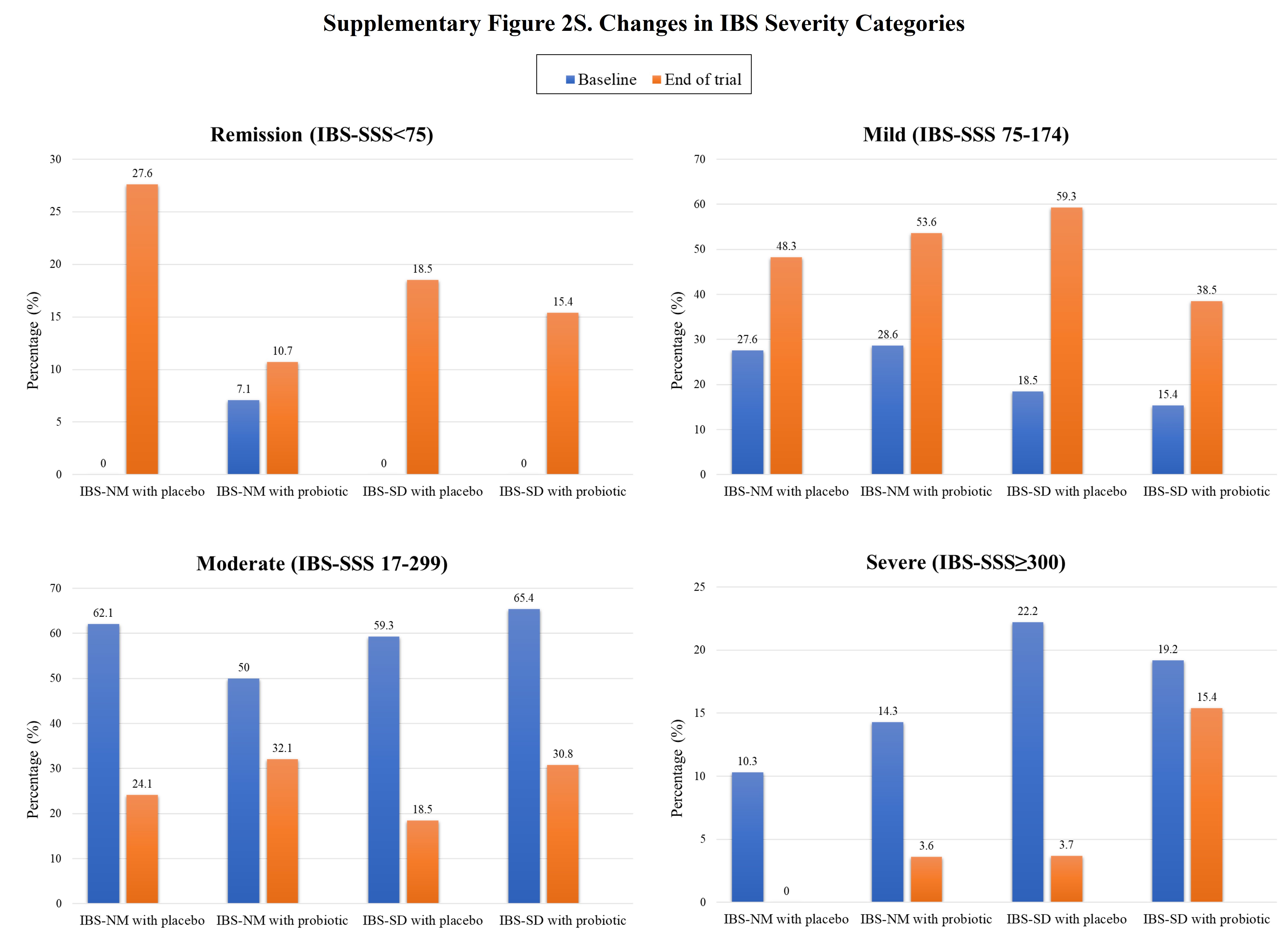

Supplement: Supplementary file 13 — Supplementary Figure 2. [file 41598_2024_60029_MOESM13_ESM.jpg]
