# Supplementary material for: A randomized, double-blinded, placebo-controlled clinical trial on Lactobacillus-containing cultured milk drink as adjuvant therapy for depression in irritable bowel syndrome
Source: Sci Rep. 2024 Apr 25;14:9478. doi: 10.1038/s41598-024-60029-2 (PMC11043363; doi:10.1038/s41598-024-60029-2)
Supplement: Supplementary file 14 — Supplementary Information 14. [file 41598_2024_60029_MOESM14_ESM.docx]

**Supplementary Formula 1S.** Sample size calculation formula.

| $n = \frac{\left( \sigma_{1}^{2}+\sigma_{2}^{2} \right){(z_{1-\frac{\alpha}{2}}+z_{1-\beta})}^{2}}{\left\vert\mu1-\mu2 \right\vert^{2}}$ |
| --- |
|  |
| $n = \frac{\left( {5.73}^{2}+{9.81}^{2} \right){(1.96+0.84)}^{2}}{\left\vert11.1-4.0 \right\vert^{2}}$ |
|  |
| $n = \frac{\left( 32.8329+96.2361 \right){(2.8)}^{2}}{{7.1}^{2}}$ |
|  |
| $n = 20.073$ |

Where σ1 and σ2 represent the pooled standard deviation of population 1 (received probiotics) and population 2 (without probiotics), respectively. μ1 and μ2 represent mean difference of population 1 (received probiotics) and population 2 (without probiotics), respectively.
